# A bacterial genome assembly and annotation laboratory using a virtual machine

**DOI:** 10.1002/bmb.21720

**Published:** 2023-03-03

**Authors:** Ellina Trofimova, Shahla Asgharzadeh Kangachar, Karen D. Weynberg, Robert D. Willows, Paul R. Jaschke

**Affiliations:** ^1^ School of Natural Sciences Macquarie University Sydney New South Wales Australia; ^2^ ARC Centre of Excellence in Synthetic Biology Macquarie University Sydney Australia; ^3^ Australian Centre for Ecogenomics, School of Chemistry and Molecular Biosciences University of Queensland Brisbane Queensland Australia

**Keywords:** bacteriophage, bacterial immunity, genome annotation, phage therapy, UPEC

## Abstract

With the global increase of infections caused by antibiotic‐resistant bacterial strains, there is an urgent need for new methods of tackling the issue. Genomic analysis of bacterial strains can help to understand their virulence and antibiotic resistance profile. Bioinformatic skills are in great demand across the biological sciences. We designed a workshop that allows university students to learn the process of genome assembly using command‐line tools within a virtual machine on a Linux operating system. We use Illumina and Nanopore short and long‐read raw sequences to reveal the advantages and disadvantages of short, long, and hybrid assembly methods. The workshop teaches how to assess read and assembly quality, perform genome annotation, and analyze pathogenicity, antibiotic and phage resistance. The workshop is intended for a five‐week teaching period and is concluded by a student poster presentation assessment.

**Abbreviations**
AMR: antimicrobial resistance
APEC: avian pathogenic *Escherichia coli*
BLAST: basic local alignment search tool.
CLI: command line interface
EHEC: enterohemorrhagic *Escherichia coli*
EPEC: enteropathogenic *Escherichia coli*
ETEC: enterotoxigenic *Escherichia coli*
NGS: next‐generation sequencing
NMEC: neonatal meningitis‐causing *Escherichia coli*
SEPEC: human sepsis‐associated *Escherichia coli*
UPEC: uropathogenic *Escherichia coli*
VM: virtual machine

## 1 INTRODUCTION

With the cost of DNA sequencing falling exponentially for over 20 years, the tools of genomics have become universal across many areas of biological science. Starting from first‐generation Sanger sequencing technology in 1977, followed by second‐generation sequencing technologies (also called next‐generation sequencing (NGS)) that came online in the early 2000 s began to cut the cost of sequencing dramatically. With the recent advent of third‐generation sequencing technologies by Oxford Nanopore and Pacific Biosciences, real‐time long‐read sequencing has become common.^1^

In parallel with the decline in cost and increase in sequencing quality, DNA sequencing and analysis have permeated popular culture with broad public knowledge and acceptance in police forensics,^2^ prenatal testing,^3^ and consumer genetic testing to assess risk of disease(s) and to assess a person's ancestry.^4^ Discussion of genome variants of the SARS‐CoV‐2 virus on the news and social media has also become common over the last 2 years during the COVID‐19 pandemic.

Equally important, but not currently appreciated as urgent as the COVID‐19 pandemic, is the threat of antimicrobial resistant (AMR) bacterial pathogens.^5^ The threat to humans and livestock from AMR pathogens is growing yearly, and options for treating antibiotic‐resistant bacteria are not increasing as quickly as needed to keep pace. The field of phage therapy seeks to treat AMR infections using viruses that infect bacteria, called bacteriophages.^6^ The bacteriophage can be used alone, or in concert with other therapies, such as antibiotics, but relies on testing large banks of phage against cultured bacteria to test for susceptibility before being deployed.^6^ High‐throughput methods to understand how bacterial hosts become resistant to phage are currently making good progress in this area.^7^ Phage engineering is focused on creating enhanced phages that may be able to infect a broader range of hosts or actively mount counter‐defenses against host defense systems.^8^, ^9^, ^10^

Understanding and being able to predict which bacteria were susceptible to which phage (host range) from bacterial genomic sequences would be incredibly important for advancing the field of phage therapy. To make raw bacterial sequencing data meaningful, we need to assemble the individual reads into larger contigs and chromosome‐scale genomes. To do that, sophisticated computational algorithms and tools have been developed.^11^ However, for many undergraduate students and research scientists, the array of tools is bewildering, and the implementation requires unfamiliar operating systems (Linux) and tools that only work from direct text input (the command line) rather than a familiar graphical user interface. Together, these factors create a significant barrier to entry for those interested in analyzing genomic sequences and can leave even experienced scientists feeling frustrated.

To overcome these issues, we designed a computational laboratory exercise as part of a suite of labs offered in a course on genomics. The genome assembly and annotation laboratory serves several teaching purposes within the course. First, the laboratory teaches procedural knowledge needed to perform genome assembly and annotation using command‐line interface (CLI) tools. We have designed the course to teach this procedural knowledge because these skills will be useful in a range of careers the students may undertake in the future. For example, the knowledge of how to perform sequential operations on DNA sequencing reads on the CLI could also be used in several research labs within our department if the students pursue further studies. More broadly, coding skills are in demand across many different aspects of biological research and will only be more critical in the future.

The sequence reads used in this exercise were generated in the course of our lab's work to understand the host range of PhiX174 bacteriophage.^12^, ^13^ While sequencing new PhiX174 host strains, we realized that some of them had characteristics of pathogenic strains, as well as potentially containing antibiotic resistance genes. Furthermore, there were signatures of defense islands potentially encoding anti‐phage resistance mechanisms.^14^

We designed a five‐week computational workshop for students to learn how to use computational tools to go from raw sequencing reads to assembled contigs, followed by structural and functional annotation, and the use of specialized annotation tools to address whether a bacterial strain is pathogenic, antibiotic‐resistant, or phage‐resistant.

We teach the genome assembly and annotation laboratory in weeks three to seven of our course, following 2 weeks of using genome browsers to explore existing genome sequences and annotations in online databases such as the NCBI GenBank^15^ and EMBL‐EBI Ensembl.^16^ In this way, we are able to explicitly link the process of de novo assembly and annotation of raw sequencing reads with prior knowledge of sequencing technologies and exploring genome sequences and annotations. Moreover, the genome sequence the students assemble and analyze using specialized tools yields results with a level of ambiguity that challenges the students to construct arguments for the presence, absence and function of a range of phenotypic characteristics without an obvious ‘right answer’. It ensures that assessments on the material cover a range of learning outcomes and the students receive a learning experience that is very similar to that found in research laboratories.

## 2 PRACTICAL ISSUES OF THE COURSE

**FIGURE 1:**
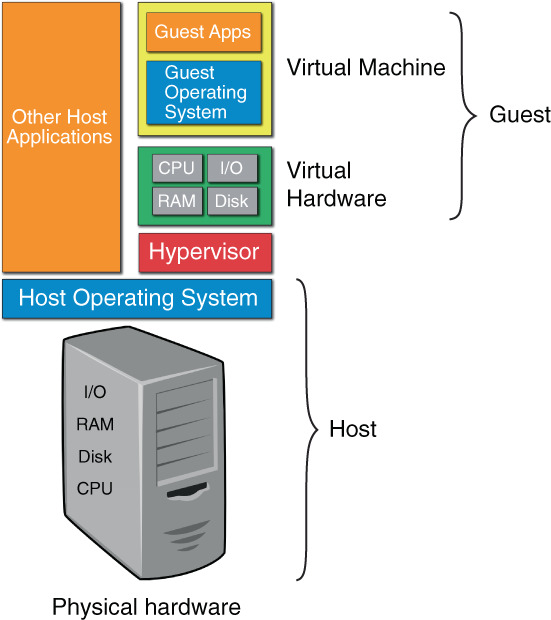
Virtual machine relationship to hardware and software. The Host machine has physical hardware consisting at a minimum of a central processing unit (CPU), a disk that can store and retrieve information, the random access memory (RAM) used to temporarily store data moving between the CPU and disk, and the input/output (I/O) system for moving information back and forth from peripherals (mouse, keyboard, and monitor) to CPU. The Host operating system (usually Windows or macOS) runs on the host computer is software that manages hardware and software resources and provides common services for computer programs. The hypervisor is a program running on the Host operating system that creates and runs the virtual machines. The virtual machine is called a Guest, and the Host and the Guest are separate and cannot directly interact unless sharing is set up. The Host machine has to share resources (RAM, CPUs, disk space, I/O), through the hypervisor, with the Guest. The hypervisor generates virtual hardware that the guest operating system and applications (apps) run on.

The workshop is part of a course that covers knowledge and techniques for both reading genomic sequences as well as writing sequences (synthetic biology). The course pre‐requisites include molecular biology, biochemistry, and cell biology courses. The analytical skills taught here are the use of a suite of computational tools to process, assemble, and annotate sequencing reads from second and third‐generation NGS technologies. All of the computational procedures for the laboratory are performed within a virtual machine (VM) running on a host computer (Figure 1). Unlike other courses, where analysis is done on local servers^17^ or the cloud,^18^ we chose to deploy the course using a virtual machine for several reasons. Working on a local computer enables the course to be performed even in the absence of an internet connection, which could be a benefit for low‐resource teaching environments. Using a cloud service can feel remote or very foreign and uncomfortable to students, whereas the experience of using a local computer is more familiar, even if the operating system is different. Further, the experience the students gain using another operating system (Linux) can be directly translated to their own personal computer if desired. For the instructor, the creation of a consistent environment to run the command‐line interface (CLI) tools ensures that they are able to troubleshoot problems across all students, using consistent and standardized strategies, without the need to customize procedures for each student's different operating systems. Additionally, the CLI tools that are used in the laboratory require a significant amount of skill and time to install and set up correctly and would be difficult to accomplish during laboratory time. Lastly, the VM is available to the students to use on their own machines if they want to pursue additional work after the course is completed. For the very same reasons, we have previously developed a VM for performing advanced machine learning work to enable research scientists to design overlapping genes^19^, ^20^ without the need for loading all the complex applications on their own computers, or the need to create a Linux partition if they do not already run this operating system.

During the course of the computational labs, students will learn to use both the Linux operating system (Ubuntu distribution) as well as the Bash programming language. Learning about the Linux operating system is important for students because it is a leading free, high‐security operating system, with low system resource requirements. It is used by nearly all bioinformatics labs around the world, ensuring tools that the students use in the computational labs will be maintained for years to come. Learning Bash is essential for students because it is a fundamental programming language that offers a fast and powerful way to directly manipulate files and data on a computer. As just one example, many sequence files are so large they cannot be fully opened on even high‐end computers because of memory constraints, but with a Linux based Bash terminal, it is easy to take a peek into the file to get the information required without overwhelming the system. Bash can also promote an interest in learning higher‐level interpreted languages like Python or R and many cloud services that are accessed and connected through CLIs.

## 3 OVERVIEW OF THE PROCEDURE

Currently, we offer the computational laboratory in the following form. The framework of the procedures is such that they can be easily modified to suit different genome sequences and learning aims as required. For example, if the students are already proficient with CLIs, week 1 material can be omitted; if the course has a learning outcome focused on horizontal gene transfer, specialized tools to study this could be used instead. The detailed procedures are outlined in Supporting Information.

The laboratory starts with setting up the virtual machine, which may or may not already be on the computers. We use a university wet lab which also contains desktop computers that the students can work at, and university IT services pre‐deploy the virtual machines. In areas with inadequate internet, or local network resources, the virtual machine can be deployed using 32GB USB sticks (currently costing less than $7USD each).

We use the excellent Software Carpentry Unix Shell Activity^21^ available online to scaffold the workshop activities. The workshop begins with the instructor giving a short lecture to describe VMs and how they operate. They then lead the students through activities to teach them how to use the Unix Shell Bash CLI (Table 1). If the students cannot finish the activity in the 4‐h workshop, they are expected to complete the activities on their own time before the following week's workshop.

**TABLE 1:** Genome assembly and annotation workshop schedule

Workshop week	Workshop activities
1	1. Setup and configure the virtual machine 2. Software Carpentry Unix Shell Activity
2	1. Short read (Illumina) quality evaluation with FastQC 2. Short read trimming with Trimmomatic 3. Re‐evaluation with FastQC 4. Short read assembly using Minia 5. Short read assembly quality assessment using QUAST 6. Long read (Nanopore) assembly using Raven 7. Long read Assembly quality assessment using QUAST
3	1. Hybrid short and long read assembly using SPAdes 2. Hybrid assembly quality assessment and comparison to Minia and Raven assemblies using QUAST 3. Mapping reads back to hybrid assembly using minimap2 4. Visualizing the mapped reads using gap5 5. Genome annotation using Prokka 6. Genome exploration using Artemis
4	1. Analysis of the bacterial strain assembly in FASTA format with: (a) In Silico Clermont Phylotyper (b) SerotypeFinder (c) VirulenceFinder (d) VFanalyzer (e) ResFinder (f) Restriction‐ModificationFinder (g) Prokaryotic Antiviral Defense LOCator (PADLOC) 2. How to create a science poster presentation
5	Week off to work on posters
6	Poster presentation

**FIGURE 2:**
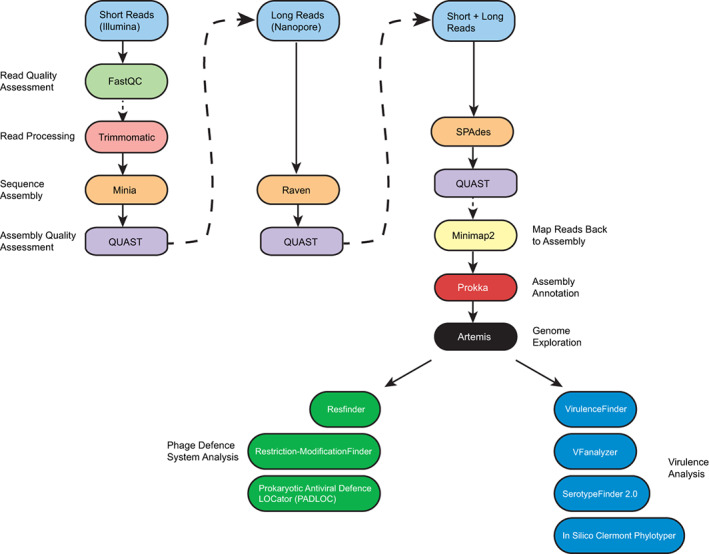
Computational tool workflow. The sequence of analyses is shown by arrows with solid arrows denoting directly using the results of one computation in the next tool and dashed arrows showing only the order of tools used, not data dependency.^22^, ^23^, ^25^, ^26^, ^27^, ^30^, ^31^, ^32^, ^33^, ^34^, ^35^, ^36^, ^37^, ^38^, ^46^, ^47^

In Week 2 the students are presented with a structured general description of Bash commands and how some require options (also known as flags) and how some do not. Additionally, specific examples from the previous week's activities are presented and broken down to show the students how the different components of commands are required for the command to function. Additionally, the concepts of next‐generation sequencing, sequence quality assessments, file formats, and fundamentals of sequence assembly are presented for a second time in a complementary way. The concepts were first presented in the lecture component of the course. The students are then presented with the background scenario and introduced to the set of experiments they will perform (Figure 2) to address the question of determining whether the bacterial strain containing the sequenced genome is pathogenic, antibiotic‐resistant, or phage‐resistant.

The students are then given raw Illumina and Nanopore sequencing reads in the form of gz compressed fastq files for the bacterial strain under consideration, and analyses are performed (Table 1 and Figure 2). Trimmomatic^22^ is used to trim low‐quality parts of sequences and remove adapter sequences. Students had previously been taught in detail about Illumina adapter sequences during lectures. Thus, this activity is using a practical application to reinforce previously acquired theoretical knowledge. The tool FastQC^23^ is used to show changes to the quality of the sequencing reads before and after trimming.

By the end of the laboratory session, students will have seen that sequence assembly using only short reads and the program Minia^24^ results in many short contigs but the higher depth and fewer indels and errors/SNPs in the contigs upon assembly quality analysis using QUAST,^25^ whereas assembly using the program Raven^26^ and only long reads results in longer contigs with more indels and higher error rate. The main assembly quality assessment parameters (L50, N50, and coverage) that the students use to determine assembly quality reinforce the theoretical knowledge of these terms that were previously taught in the lecture component of the course.

In Week 3, the students are presented with a review of the previous week's work, along with information about hybrid sequence assembly methods that use both short and long reads. The Week 3 activities (Table 1) involve creating a sequence assembly using SPAdes^27^ and assessing its quality with the tool QUAST, followed by mapping reads back to the assembly to determine if any regions show unusual patterns. We have found the SPAdes tool requires more RAM than most laptop computers can deliver. If student computers are to be used for the workshop, then either the Wengen hybrid assembler^28^ should be used in place of SPAdes, or the instructor can skip the computational step of the hybrid assembly and instead provide students with the resulting files for downstream analysis.

The genome assembly does not create a closed assembly, and even with the hybrid assembly method, there are 44 contigs generated. The Minia, Raven, and SPAdes assemblies are compared using QUAST to highlight strengths and weaknesses between the different sequencing technologies. This result is related back to material presented in lecture, showing that most model organism genome sequences are still not complete (although the first full human genome sequence was completed during the most recent delivery of this course^29^), and relate this information to N50 and L50 measurements of assemblies. The contigs of the assembly are then annotated for protein coding sequences, tRNA, and small RNAs using the tool Prokka.^30^ The annotated genome is then explored briefly using Artemis^31^ to gain proficiency with the tool. Some of the smaller contigs are plasmids and these may be a focus of study if desired for course learning outcomes.

In Week 4, the activities of the previous week are reviewed, and each group of two students are tasked with using the assembled annotated genome sequence to address one of two questions (randomly assigned). The first problem is to determine if the sequence indicates the bacterium is pathogenic; the second problem is to determine if the bacterium is antibiotic‐resistant and whether it is resistant to any bacteriophage that might be used in phage therapy. The students then work through analyzing the sequence using specialized web‐based tools^32^, ^33^, ^34^, ^35^, ^36^, ^37^, ^38^ (Table 1 and Figure 2) with the instructor's guidance.

In week 5, the students are given off to work on their poster and practice their presentation, and in week 6 the laboratory is a poster session. In previous offerings, we have had half the students present in one session and the other half in a second session which enables each half of the students to participate as poster evaluators as well as presenters, giving them a diverse perspective from both sides of the presentation.

## 4 ASSESSMENT OF STUDENT LEARNING

The genome assembly workshop learning objectives were:

1. Recall from lecture the sequence format types and Phred quality scores.
2. Recall from lecture the steps in genome sequencing and assembly.
3. Define and describe measures of genome assembly quality, including L50, N50, and contiguity.
4. Compare second and third‐generation sequencing (next‐generation sequencing) technologies and their advantages and disadvantages.
5. Discuss and relate student laboratory exercises to ‘real‐world’ commercial and academic uses of DNA sequencing and analysis.
6. Demonstrate proficiency with specialized DNA sequence analysis.
7. Record experimental procedures performed, and identify any deviations from standard protocol.
8. Record data from bioinformatics experiments.
9. Demonstrate proficiency with Bash command‐line interface and Ubuntu Linux.
10. Employ graphics design software to create figures and a scientific poster.
11. Report genome sequencing, annotation, and specialized analysis results within the context of experimental objectives.

During the process of practicing these technical aspects of DNA sequence analysis, the students were also learning about several important aspects of bacterial biology. These concepts include:

1. Genome structure
2. Genetic networks
3. Antibiotic resistance mechanisms
4. Virulence mechanisms
5. Antiviral defense systems

Student understanding of the specific material being taught in each workshop is subjected to regular formative assessment, including anonymous polls and the muddiest point activities. Moreover, before covering any material in the Week 1 workshop, a background knowledge probe^39^ is given to students through a cloud‐based form to understand the level of prior knowledge of the material. This probes their level of understanding of genomics, computers, command‐line interfaces, and Linux. Following the conclusion of the poster session, another background knowledge probe is administered to the students to assess the change in knowledge over the workshop.

Student understanding of the major concepts of the computational experiments to assemble, annotate, and assess virulence and resistance to phage was evaluated through a scientific poster session summative assessment. The poster is A3 paper size and is presented during an in‐class poster session. During various offerings of the course, we have had students either print A3 size physical posters or just present them on a 1080p or greater size monitor in digital format, which is approximately the same size as an A3 paper (11–3/4 x 16–1/2 inch; 297 x 420 mm). Depending on resources available to the instructor, this can be varied however they desire.

The session features a short (~5 min) oral explanation of the poster to two examiners, followed by the examiners asking questions to the presenters about the poster content for 5–10 min. Questions included those designed to assess student understanding of the objective of the experiment, how the experiment was performed, and the meaning of the results within the context of the field(s) of interest (phage therapy, antibiotic resistance, and virulence). Some example questions included: “Describe the objective of this experiment”, “explain how the result of method X shows that the bacterium may be virulent”, “based on your analyses, what type of pathogenic *E. coli* (EHEC, ETEC, EPEC, NMEC, SEPEC, APEC, UPEC, etc.) would this bacterium be most similar to?”, and “what do your results mean within the context of using genome sequencing to (1) determine bacterial phage susceptibility/resistance or (2) determine bacterial virulence.”

The instructor's assessment included using a detailed rubric to assess how the poster met the grade criteria across five categories: (1) Knowledge and Understanding; (2) Writing Quality; (3) Visual Presentation; (4) Figures, Diagrams, and Tables; and (5) Referencing. In addition, students' answers to questions were assessed for their depth and breadth of knowledge, and their ability to synthesize ideas across the different areas of their analysis.

Student assessment also included self‐assessment and peer‐assessment. Students were asked to rate their performance in the group work and to identify one area of the analysis they felt their contributions were most effective, and one aspect where they struggled to effectively contribute. All students individually evaluated at least two posters from other groups. These assessments were also a valuable learning experience as the students were able to see other ways of constructing a poster on a similar topic as their own, as well as learn about the topic they were not assigned (e.g. those making a poster analyzing virulence were also evaluating student posters who were analyzing antibiotic and phage resistance).

**FIGURE 3:**
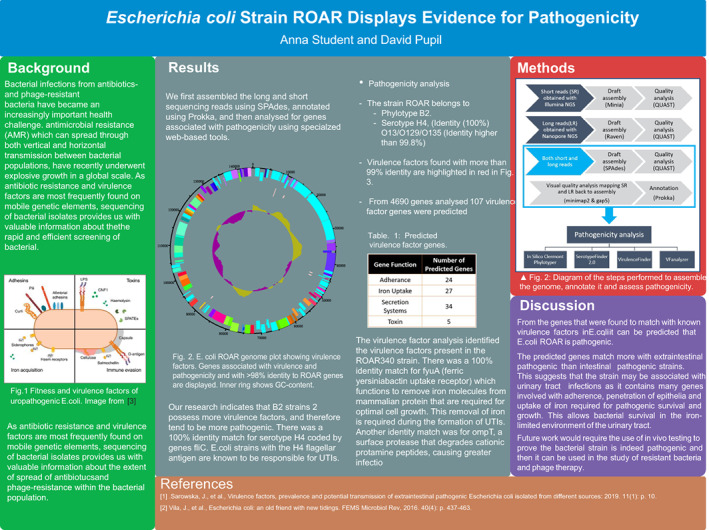
Representative student poster. Student analysis to determine if bacterial genome encoded genes are associated with pathogenicity.

The posters commonly included the main sections of Background, Methods, Results, Discussion, and References. Between 1–4 figures and 1–2 tables were also used. The top‐scoring posters tended to have more figures and tables, but this was not exclusively the case. A representative poster is shown in Figure 3. The student analysis of the genomes showed they were able to successfully identify a range of genes associated with pathogenicity. The main challenge with answering this biological question was a matter of categorizing and synthesizing the functions and pathways the different genes are involved in into a cohesive picture. Many of the tools reported the ‘hits’ between genes in the bacterial genome under consideration and known pathogen‐associated genes, with the strength of the hit represented by e‐values and % identity. In evaluating these hits, the students were able to draw on knowledge and skills developed in the first workshop series of the course, where they performed BLAST analyses and evaluated the e‐value, % identity, and % coverage of the hits. The challenge for the groups analyzing antibiotic resistance and phage resistance genes revolved around doing a deeper analysis of fewer hits, with a detailed explanation of restriction‐modification systems and a description of a putative anti‐phage system identified.

Several common student pitfalls during the computational laboratory were noted. One of the issues disrupting the genome assembly and analysis was not following the CLI command sequence. With some steps omitted, students missed output files required as input files for the subsequent commands and could not proceed further. Another problem was copying commands from the workshop PDF or Word file with additional spaces that disrupted the command execution. In addition to that, some students experienced troubles navigating through different directories with raw sequence, assembly files, and quality reports. We found the most challenging conceptual part of the workshop was that the students were required to work with a novel *E. coli* strain that was not fully described in the previous literature. Therefore, they had to conduct the investigation by themselves, relying only on publications with similar data on *E. coli*, and could not compare their interim results of assembly and analysis with any available data. Additionally, web tools used for the analysis tend to get updated frequently, resulting in non‐working websites or new data on bacterial strains that have to be taken into consideration for poster preparation.

## 5 EDUCATIONAL IMPACT

The positive educational impact of this laboratory exercise was clearly shown by the outcomes of knowledge probes performed before and after the workshop, and the quality of the scientific poster sessions. Students came into the workshop with the vast majority of them never having used command‐line tools, Linux operating systems, or having performed any sequence processing or assembly. The results of background knowledge probes revealed that the students had gained enough knowledge that they could now understand questions in the areas of genomics and genome assembly and annotation, and some even felt confident enough that they could teach a fellow student. The poster session resulted in students presenting their analysis of the assembled bacterial sequence to both fellow students and instructors. The majority of the posters showed that the students could use several separate pieces of evidence to support their claims of which attributes the bacterial strain possessed. The functional annotation of genes were not clear cut in many cases, and the students needed to deal with information of differing levels of quality and completeness to build up evidence. These skills are highly valuable in a range of areas in education and the workforce that require analysis and argument development.

To understand the output of the special analysis tools used in week 4, students used prior knowledge of e‐values and percent coverage previously learned in a workshop dedicated to the Basic Local Alignment Search Tool (BLAST),^40^ and genome databases (NCBI and EcoCyc^41^), thus reinforcing these important foundational bioinformatics concepts.

Aside from positive links formed between the theory and practice of DNA sequencing and assembly, the students left the workshop with procedural knowledge and skills on how to perform foundational computational methods in Bash and bioinformatics. These skills will enable the students to learn other programming languages easier, and to understand the benefits and limits of computational analyses. Moreover, the students will be well equipped to engage with reports of pathogenic bacteria, phage therapy, and the antibiotic resistance crisis, whether it is from news or scientific publications.

One potential extension to the educational impact of this workshop could be through analyzing other bacterial genomes. There are so many different bacteria that could be studied using the foundations of the workshop outlined here. For example, instead of antibiotic resistance and pathogenicity, the students could study bacteria that perform important roles in the biogeochemical cycle^42^ to illustrate concepts of bacterial metabolism and physiology.

The workshop could also be extended by tasking the students with discovering and downloading sequencing reads from the NCBI's Sequence Read Archive (SRA) and analyzing in a different way than was done by the original authors. The area of bioinformatics meta‐analysis has exploded because of the increasing abundance of freely available sequence data with sufficient attached metadata.^43^ With skilled instructor guidance the students could have the potential to make a real contribution to science.

In the area of using different sequence analysis tools, the sequence data presented in this workshop likely contains plasmids that students could identify and analyze separately using plasmidSPAdes.^44^ This could be done in week 3 (Table 1). Lastly, since this workshop does not perform a polishing step to generate a genome sequence of higher quality, this could easily be added through the use of a tool such as Pilon^45^ which would give the students more experience working with the genome sequence at the nucleotide level and present opportunities to teach material in the domain of single‐nucleotide polymorphisms and sequencing machine error rates.

In summary, this work has outlined a cost‐effective workshop for undergraduate or graduate students that teaches fundamental knowledge and procedural skills needed to perform bacterial genome sequence assembly and analysis.

## CONFLICT OF INTEREST STATEMENT

The authors declare no competing conflicts of interest.

## Supporting information


**Data S1 ‐** VirtualBox Setup + Linux Bash

**Data S2** ‐ Short and Long Read Assembly

**Data S3** ‐ Hybrid Assembly

**Data S4** ‐ Specialized Analysis
