# Supplementary material for: A bacterial genome assembly and annotation laboratory using a virtual machine
Source: Biochem Mol Biol Educ. 2023 Mar 3;51(3):276–85. doi: 10.1002/bmb.21720 (PMC10947226; doi:10.1002/bmb.21720)

Workshop 1: Virtual Machine Setup & Ubuntu Linux Bash

Before we can start assembling and analysing genomes we need to learn a few basic skills. Installing all the bioinformatic tools we will be using to assemble the genomes is time consuming and can work differently based on each computer installed on. To get around this problem we are going to use a **virtual machine**, which is like another computer running on top of your physical computer, but inside a window. The advantage of the virtual machine is that it is pre-loaded with the tools you need to use and is the same for everyone.

The operating system that is loaded on our virtual machine is called Ubuntu Linux, which is an open-source Unix-like operating system. If you’re an Apple user, the macOS is based on Unix so you may recognize some features. Bioinformaticians love Linux so we’re using it to access the tools they build to assemble and annotate genomes. It will look a bit different than the Windows or macOS you’re used to, but there should be enough common features that you’ll figure it out in no time.

What might be more difficult for you is the terminal or **shell** that we will be learning about today. This is a way to access the computer using text only, which is called the **command line**. Instead of a graphical user interface (**GUI**) you will learn text commands that give you complete control the file system and processor of your computer using a command line user interface (**CLI**). There is a bit of a learning curve to work on the command line, but once you get it you can do things with great efficiency and run scripts that are not available through GUIs.

Today, you will **work individually** so that you can gain the most benefit from hands-on practice.

**1. Installing a virtual machine using VirtualBox**

A. Install VirtualBox on your ***host*** machine (your computer). Go to: https://www.virtualbox.org/wiki/Downloads

B. Download and install the software appropriate for your host operating system (Window, macOS, or Linux).


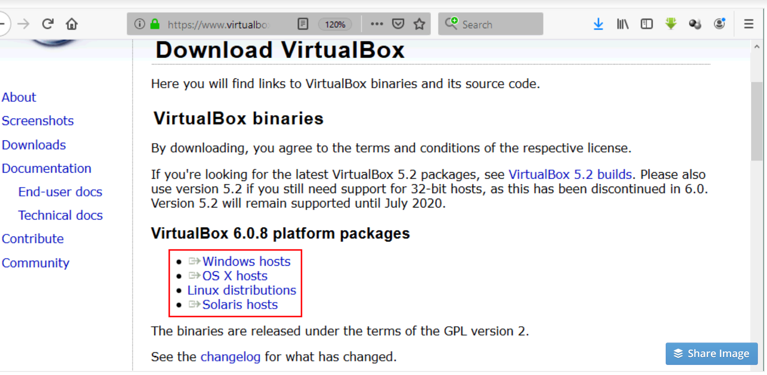


**2. Download, or transfer from a memory stick, the virtual machine onto your computer.**

The virtual machine is called genomics. The virtual machine consists of the genomics.ova file. If the virtual machine is being distributed in a zip file state to save space, the file may be genomics.zip, in which case you will need to unzip the file before using it.

**A. To download to virtual machine**

The virtual machine file is ~22GB in size so ensure you have plenty of room on your disk and a fast internet connection to download it.

The disk size of the virtual machine is 40GB**,** so as you add data to your virtual machine **ensure you have at least 65GB free on your computer's disk.**

Access the .ova file which contains the virtual machine (called genomics.ova) here:

<https://zenodo.org/record/7566978>

The starting password for the Ubuntu operating system on the virtual machine is: **mols1234**

We strongly suggest you change the password once you start using the virtual machine.

To check that the genomics.ova file downloaded correctly on **macOS**, start up a CLI such as Terminal in the directory you downloaded the file to and run:

$ md5 genomics.ova

To check that the genomics.ova file downloaded correctly on **Linux**, start up a CLI such as Terminal in the directory you downloaded the file to and run:

$ md5sum genomics.ova

To check that the genomics.ova file downloaded correctly on **Windows**, start up a CLI such as Command Prompt in the directory you downloaded the file to and run:

C:\> certutil -hashfile genomics.ova MD5

The result of these commands should be: 3c3e9b5365d8f8b505fcda89e6aa5b5b

If your result does not match, then it is likely that the download was interrupted and the genomics.ova file was corrupted. Please try to download again. In the future, if the link to this resource becomes broken, please check our lab website for details: <https://www.jaschke-lab.science/>

**B. Add the virtual machine to your VirtualBox.**

• Open VirtualBox then click on the Tools icon in the upper left of the page. This resets the view.

• To add the genomics virtual machine to VirtualBox, on the menu bar click **File->Import Appliance**


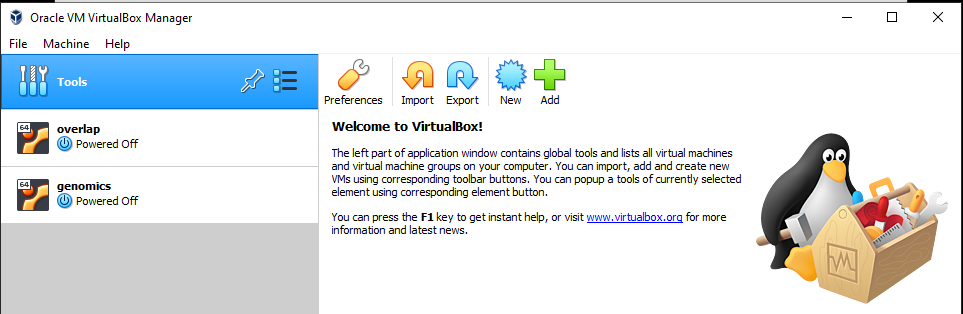


• On the new window that pops up, click on the file folder with up green arrow.

• Locate the genomics.ova file on your hard disk and click open. Follow the prompts, leaving all default options the same. Choose the location where the virtual machine will be run from, ensuring there is at least 65GB free space on this drive. The virtual machine can be run from an external USB stick but will be slower than if running from a computer disk.


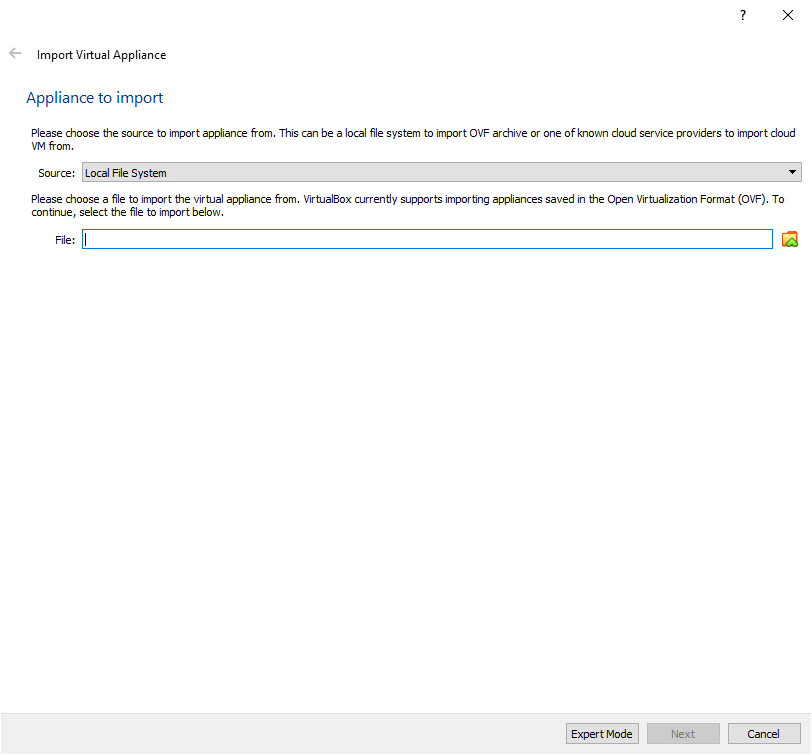


**D. Adjust the RAM and processors allocated to your virtual machine.**

• Open **Settings**

**
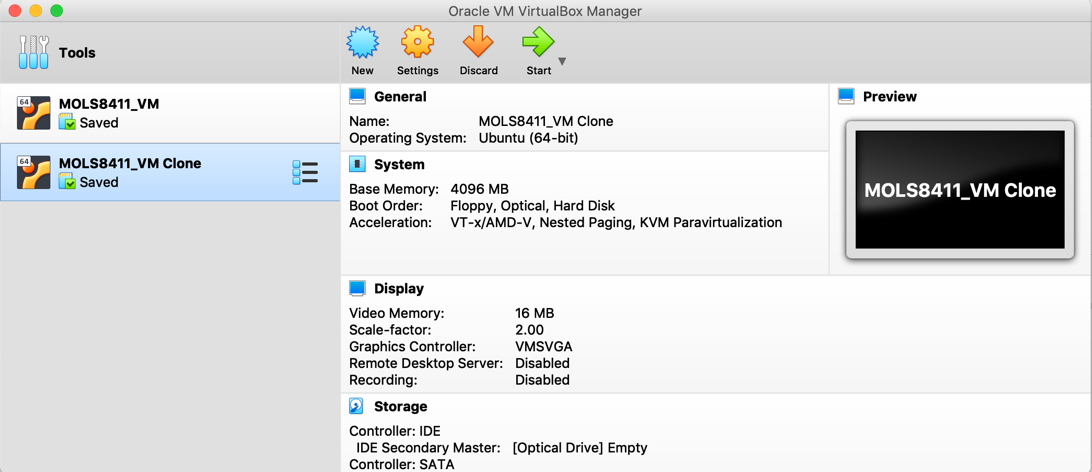
**

**Click on System>Motherboard**

Adjust within the green zone. Don’t move into orange/red or your computer will stop working and crash.

**
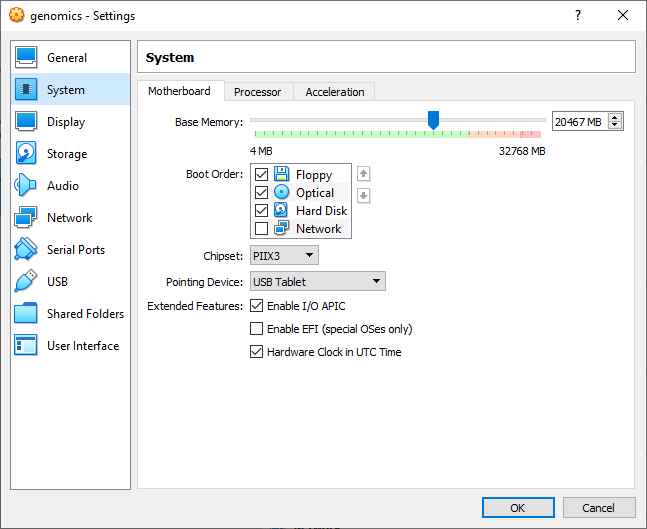
**

**Click on System>Processor**

Adjust within the green zone. Don’t move into orange/red or your computer will stop working and crash.

**
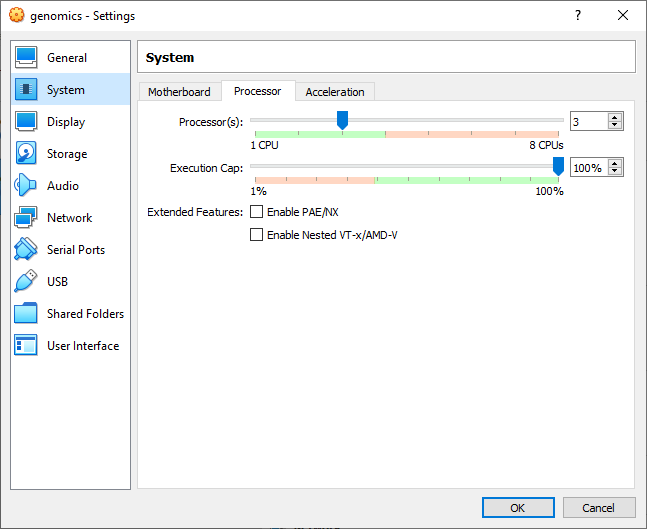
**

**2. Running your virtual machine**

Click on VirtualBox and then select the genomics virtual machine on the left. Click the Start icon (green right-facing arrow).


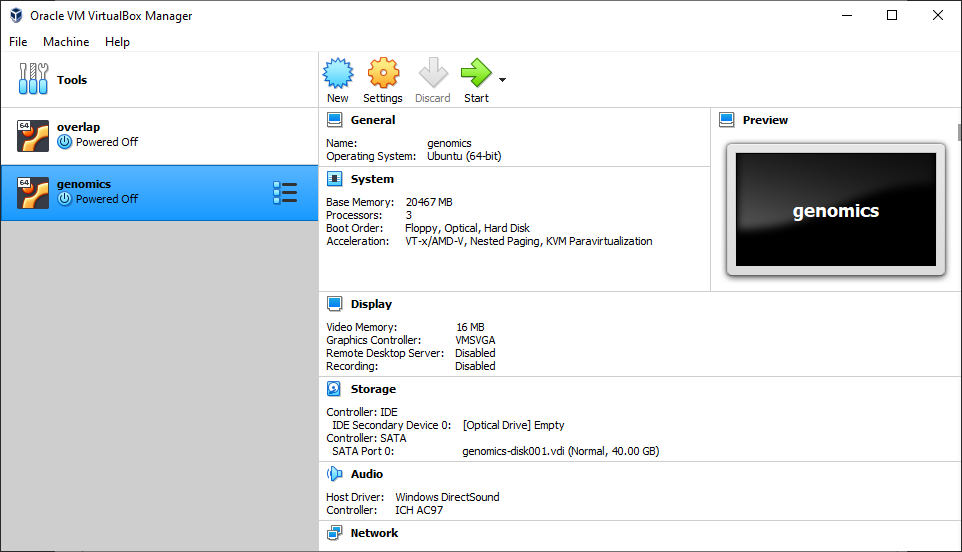


*This will boot up the virtual machine just as if you were booting up a computer. It should open a new window that is your view into the guest Ubuntu operating system.*

**3. Learning how to use the Unix Shell**

To deploy many of the software tools to assemble and annotate a genome you will need to use command line tools. Since many of you have never used these, we will spend the workshop today learning how the command line interface of Ubuntu.

**Reminder: Record Keeping**

Keeping track of your work in a notebook like OneNote, which a good way to keep records of your workflow logic and results. Your virtual machine could crash and lose all your data but if you keep track of the steps and commands you execute you can reconstruct what you’ve done and quickly recover.


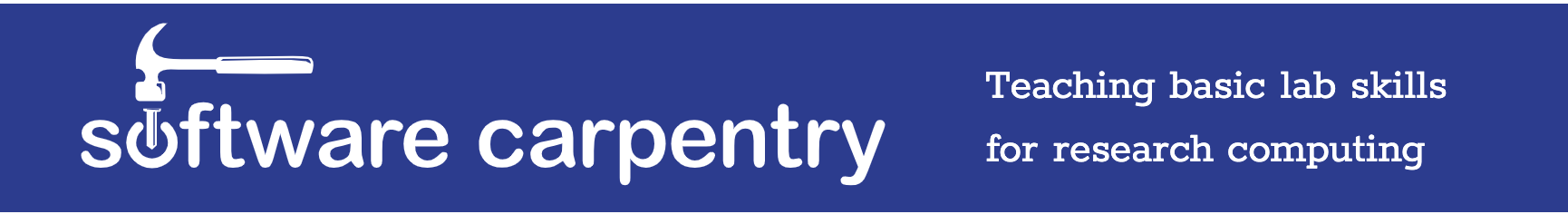


1. As outlined in the pre-workshop lecture, you will first startup **Firefox** inside the virtual machine and go to <https://swcarpentry.github.io/shell-novice/setup.html>

2. Then download the shell-lesson-data.zip file to your Downloads/ folder on the genomics virtual machine

3. Then startup your Terminal CLI.


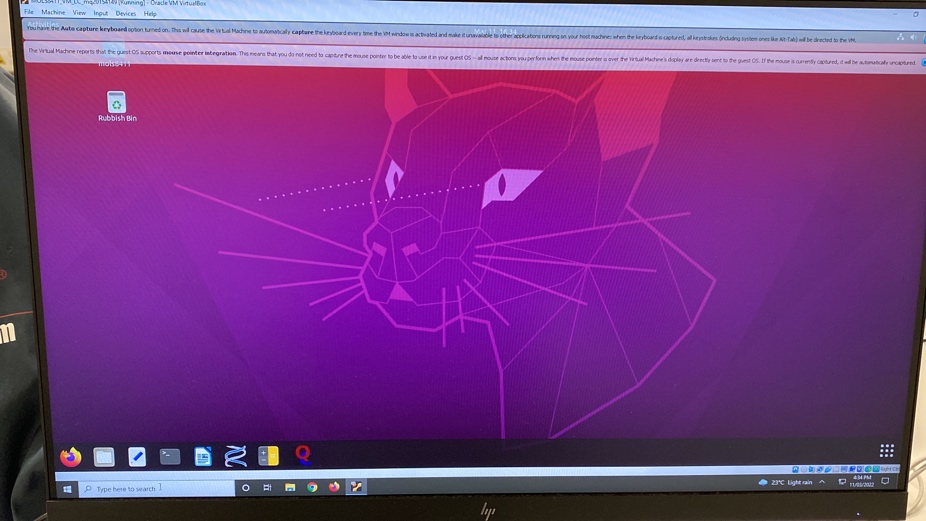


Which looks like this:

22

**
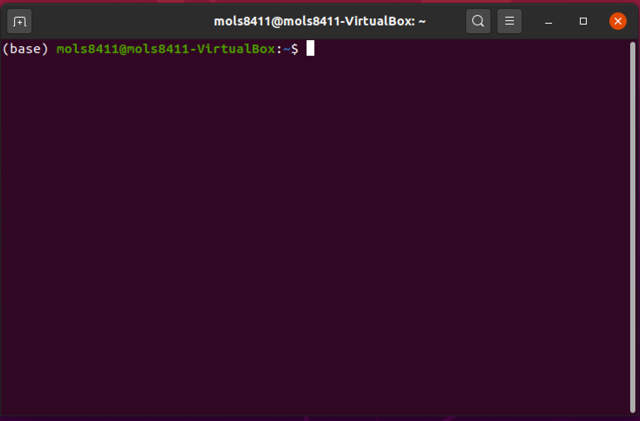
**

**4. Follow along the lessons on Software Carpentry - Unix Shell**

If you have any questions please raise your hand and it will be addressed as soon as possible.

We will go through this live now. Any remaining work that needs to be done to finish you will do on your own before next workshop.

**5. Safely shutting down your virtual machine**

The virtual machine is like a little computer running inside your physical computer and as a result it needs to be shut down properly or you will lose data and possible corrupt the (virtual) hard disk rendering it unusable.

To shut your virtual machine down safely, click the down triangle in the top right corner of the virtual machine desktop.

This will bring down a menu and you will click ‘’Power Off/ Log Out” which opens another menu and in this one click “Power Off” which will bring up another window where you either wait 60 seconds and it will power off itself or you click “Power Off” again.

**
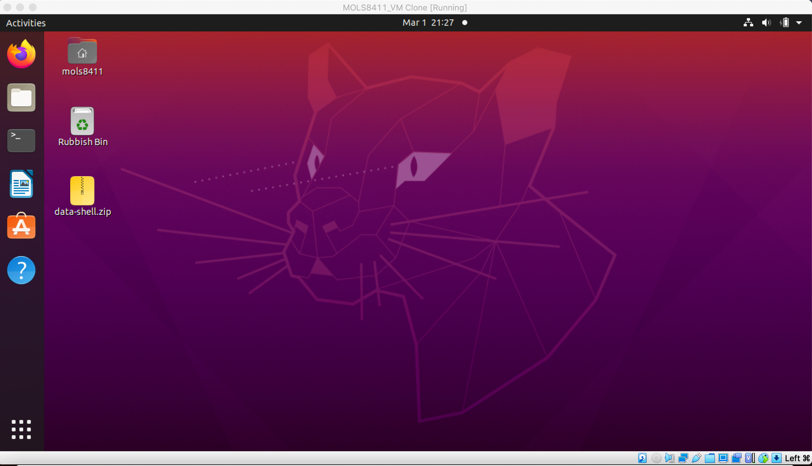
**

**
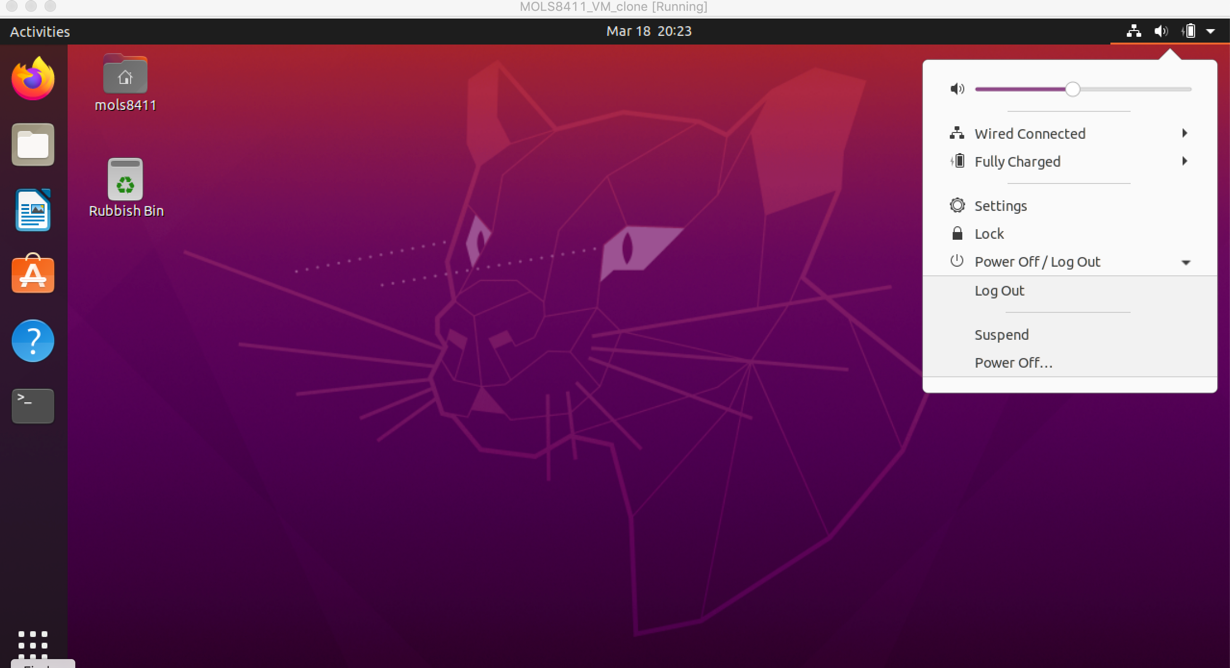
**

**
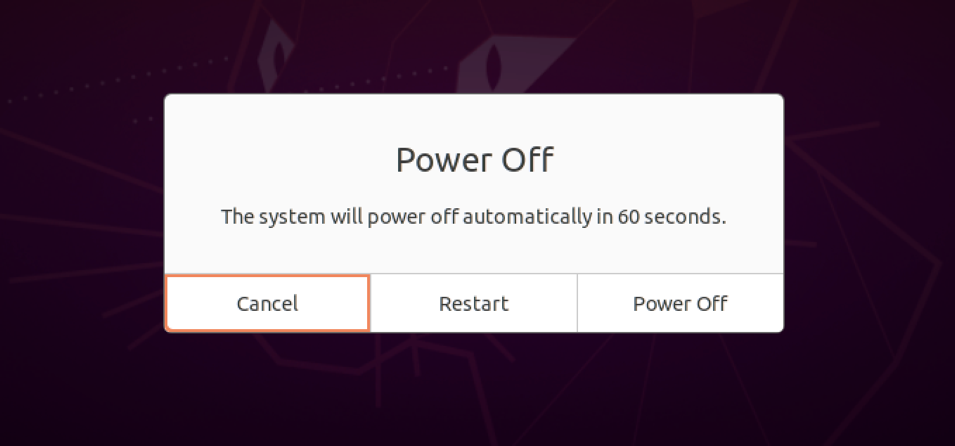
**

**6. Continuing lessons on your own machine**

**A. Continuing the lesson on a Windows machine**

• backup all the files on your computer.

**Warning**: the following involves modifying your computer in such a way that data loss is possible.

• Go here: <https://carpentries.github.io/workshop-template/#shell>

• Click on the ‘Windows’ tab and follow the directions to install Git and Bash on a Windows computer.


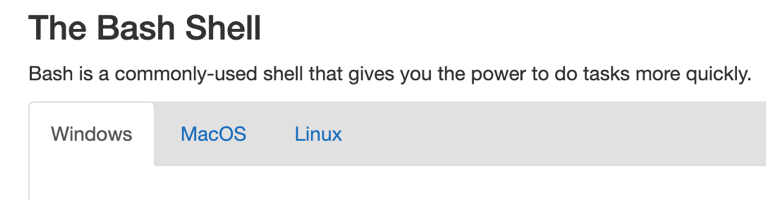


**B. Continuing the lesson on a macOS machine**

• backup all the files on your computer.

**Warning**: the following involves modifying your computer in such a way that data loss is possible.


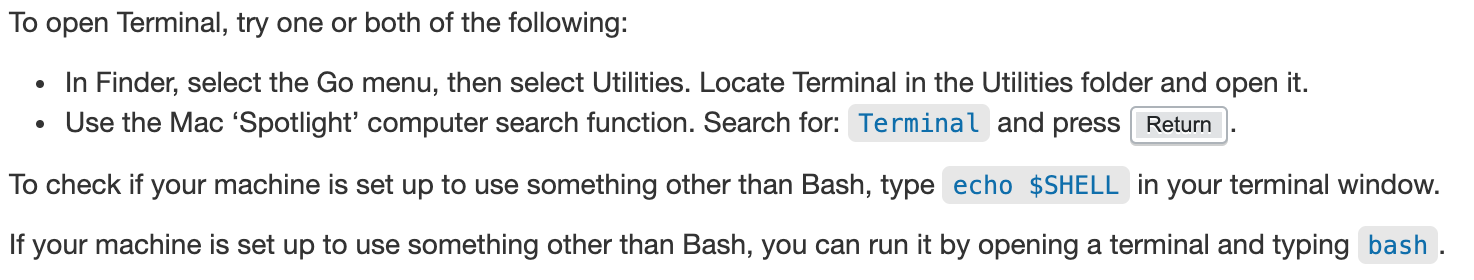

Supplement: Supplementary file 1 — Data S1 ‐ VirtualBox Setup + Linux Bash [file BMB-51-276-s004.docx]
