# Supplementary material for: A bacterial genome assembly and annotation laboratory using a virtual machine
Source: Biochem Mol Biol Educ. 2023 Mar 3;51(3):276–85. doi: 10.1002/bmb.21720 (PMC10947226; doi:10.1002/bmb.21720)
Supplement: Supplementary file 2 — Data S2 ‐ Short and Long Read Assembly [file BMB-51-276-s003.docx]

Workshop 2: Sequencing Read Quality Evaluation and Assembly Using Minia and Raven

Antibiotic-resistant bacteria are an increasing concern amongst hospitals worldwide, with many patients presenting infections that are nonresponsive to current treatments. It is estimated that by 2050 additional 10 million people per year will be dying worldwide due to antimicrobial resistance. To resolve this problem, many scientists are turning to phage therapy that uses bacterial viruses, or bacteriophages, to control pathogenic bacteria in humans, plants, and animals. In order to create more potent bacteriophages, we need to understand the interaction between bacteriophages and their hosts at the genomic level by sequencing and analysing bacterial genomes.

The sequencing revolution of the past 25 years has resulted in a deluge of sequence data, but to make that raw data meaningful we need to assemble the individual reads into larger contigs and chromosome-scale genomes. To do that, sophisticated computational algorithms and tools have been developed.

The first step in the process of assembling a draft genome sequence from raw sequencing reads is to assess the quality of the reads (Fig. 1) followed by trimming off the low-quality parts of the sequence.


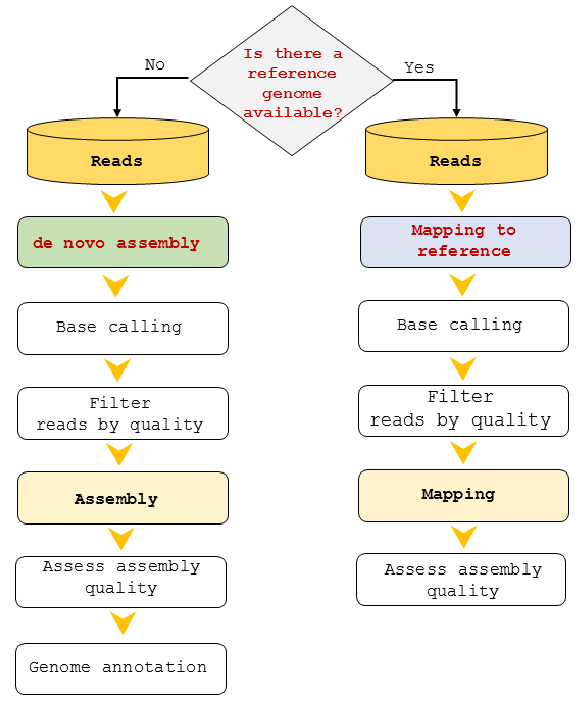

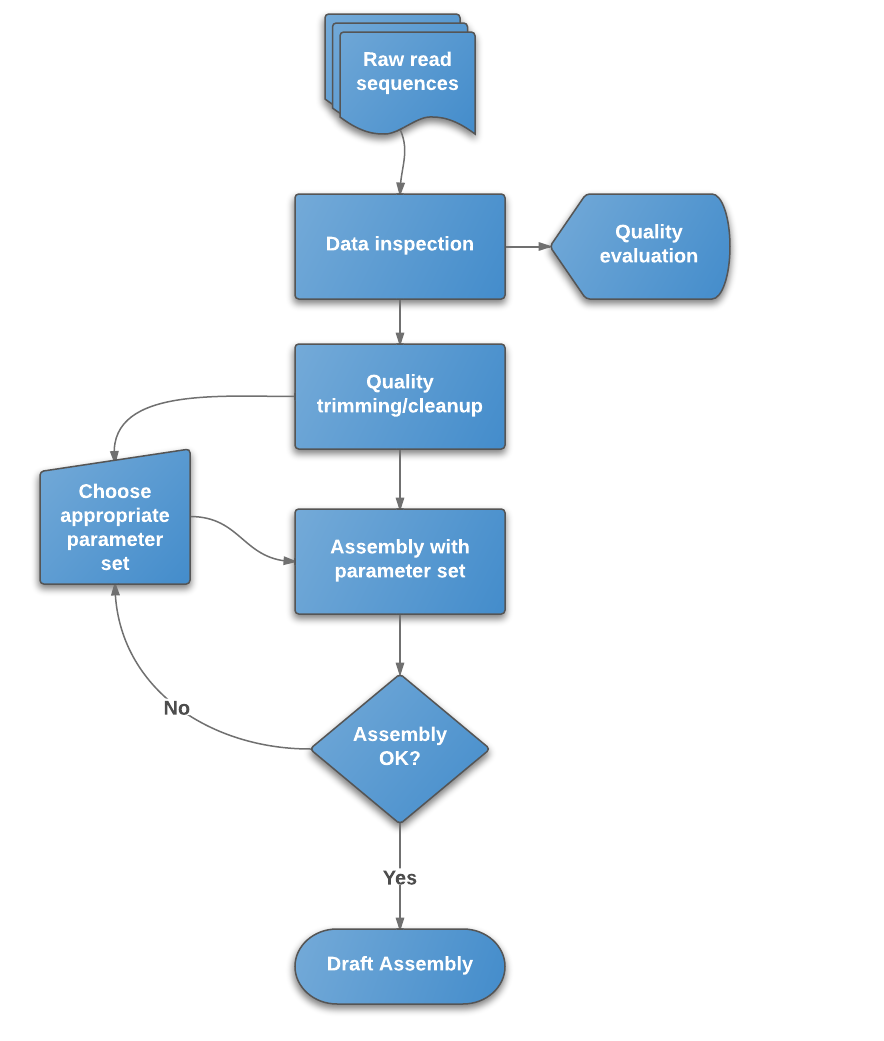


**Figure 2. Flowchart of genome assembly: de novo and based on the reference genome**. From: https://www.researchgate.net/publication/315318890_REVIEW-ARTICLE_Bioinformatics_an_overview_and_its_applications

**Figure 1. Flowchart of *de novo* genome assembly steps.**

**From https://www.melbournebioinformatics.org.au/**

In this workshop we will locate sequencing reads from two different types of sequencers, short reads from Illumina second-generation technology, and long reads from Nanopore third-generation technology. Each has advantages and disadvantages for genome assembly and we will treat each with tools specifically designed to handle the distinct type of data. Today you will be working in your normal groups of two.

**Reminder: Record Keeping**

Keep track of your work in a notebook like OneNote or in a Word file. This lets you record your workflow logic as well as the code you used to get your results. **Your virtual machine could crash and lose all your data** but if you keep track of the steps and commands you execute you can reconstruct what you’ve done and quickly recover. **You will hand in your notes as part of Assessment #2**.

**NOTE:**

Code, programs, directories, and file names will all be represented by courier font and grey background in these notes.

1. **Start your Virtual Machine**

Today, we will be doing all the analysis from inside the virtual machine genomics. Start it up using the same method as is outlined in Workshop 1.

**WARNING**: make sure you shut down the virtual machine properly when you are done for the day. See below for the detailed protocol.

1. **Get Sequencing Data**

The sequencing reads are on your virtual machine disk at these locations:

/home/genomics/roar340

The sequencing reads you will work with in this project consist of:

**(A) Short reads (sr): Illumina next-generation sequencing (NGS) files containing raw sequencing reads:**

(i) Forward direction reads (left to right on the template strand) are designated by a ‘**_1**’ suffix (ii) Reverse direction reads (right to left on the template strand) are designated by a ‘**_2**’ suffix.

sr_1.fq.gz

sr_2.fq.gz

**(B) Long reads (lr): Nanopore NGS files containing raw sequencing reads:**

(i) only in the forward direction

lr.fq.gz

**Questions to answer in your notebook:**

1. What does .fq stand for?

2. What does .gz stand for?

1. **Examining your Illumina dataset using the head command**

Let’s take a look at the Illumina read files we downloaded. To do that we need to first unzip the files.

In these notes when this code is displayed:

$ ls

What you should type into the command line interface (CLI) called Terminal is the letters 'ls' without the quotes and without the dollar sign ($). The dollar sign is to signify a CLI prompt. That is, you should type this information into the CLI so that it displays on your screen as in the notes.

- 1. **Depending on which one you have been assigned, navigate to the roar340 directory where the sequencing files are stored on your virtual machine’s disk.**

There are two ways to do this:

- - 1. **First method:**

**(A) Command Line Interface (CLI)**

Type:

$ cd

This ensures you are starting at your home directory: /home/genomics

Then execute:

$ cd /home/genomics/roar340

This takes you to the roar340/ sub-directory directly via an absolute path and would work no matter where in the file system we were. Navigating the file system using command line interface (CLI) is one of the more challenging aspects of working in CLI for many people.

Absolute path: specifying the location of a file or directory from the root directory(/).

i.e. a complete path from start of actual file system from / directory.

Relative path: the path related to the present working directory (pwd).

You could have also gone into the roar340/ sub-directory from our home directory using a relative path:

$ cd roar340

or

$ cd roar340/

**(ii) Check that the files are there:**

$ ls

which should then display:

sr_1.fq.gz

sr_2.fq.gz

lr.fq.gz

- - 1. **Second method, using the application 'Files'.**

Files is the Linux version of File Explorer on Windows and Finder on macOS. It is the way you graphically see and manipulate your files.

You should see an icon of a white folder at the bottom of the screen, click on it.

Navigate to the location the sequencing files are stored (e.g. home -> genomics-> roar340)

You should see three files:

sr_1.fq.gz

sr_2.fq.gz

lr.fq.gz

- 1. **Now, we want to unzip the files using the gzip command, but not delete the original zipped file either, because we will be using that for subsequent analyses.**

If you navigated to the directory using the CLI, please proceed below.

If you instead used the Files graphical user interface (GUI), please follow these steps to open a Terminal CLI in your current location in Files:

(i) Left click on the down triangle next to the current folder info at the top of the page

(ii) This opens a menu

(iii) Left click 'Open Terminal' and you now have a CLI Bash Terminal at the folder you are looking at in Files.


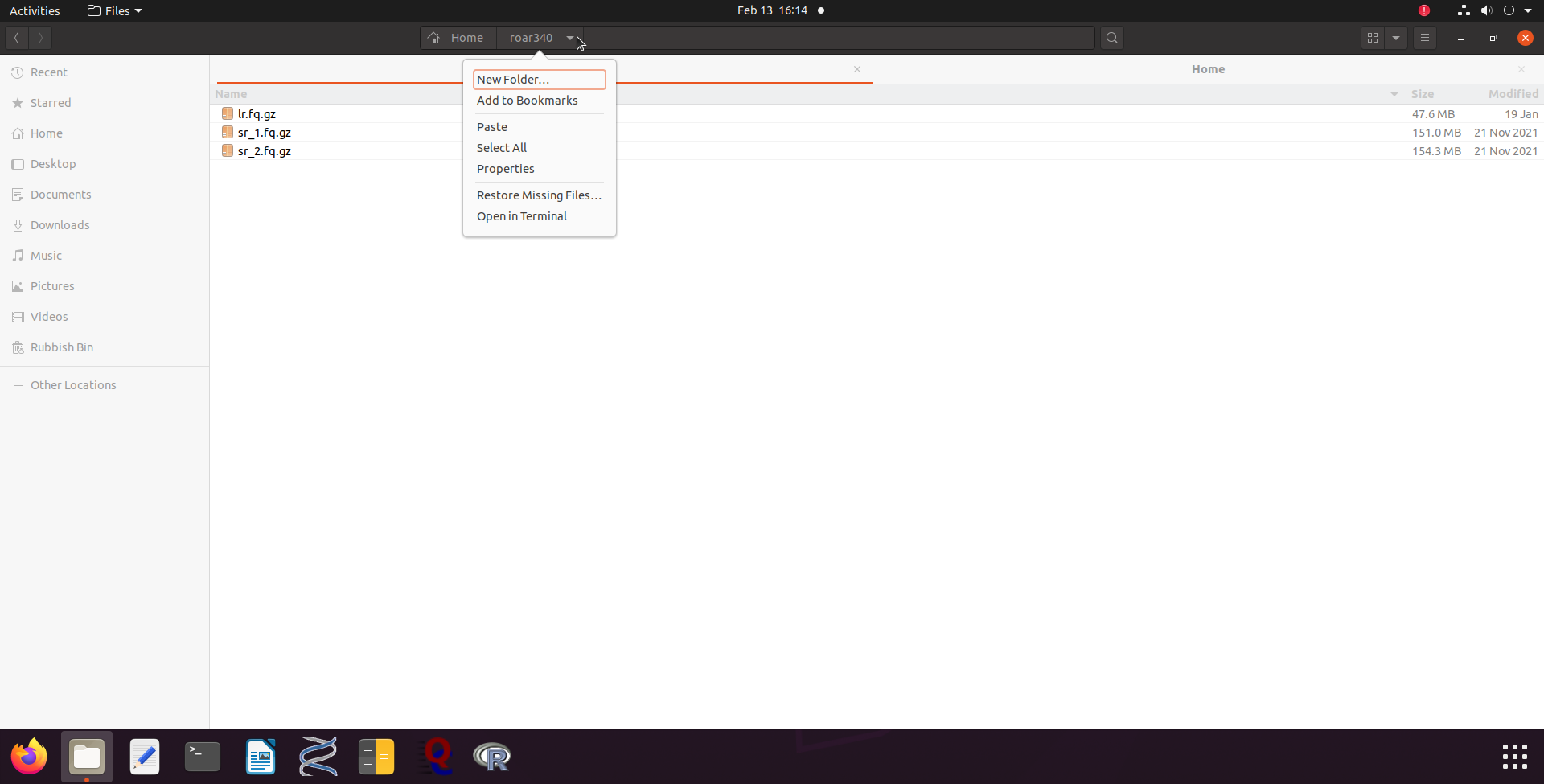


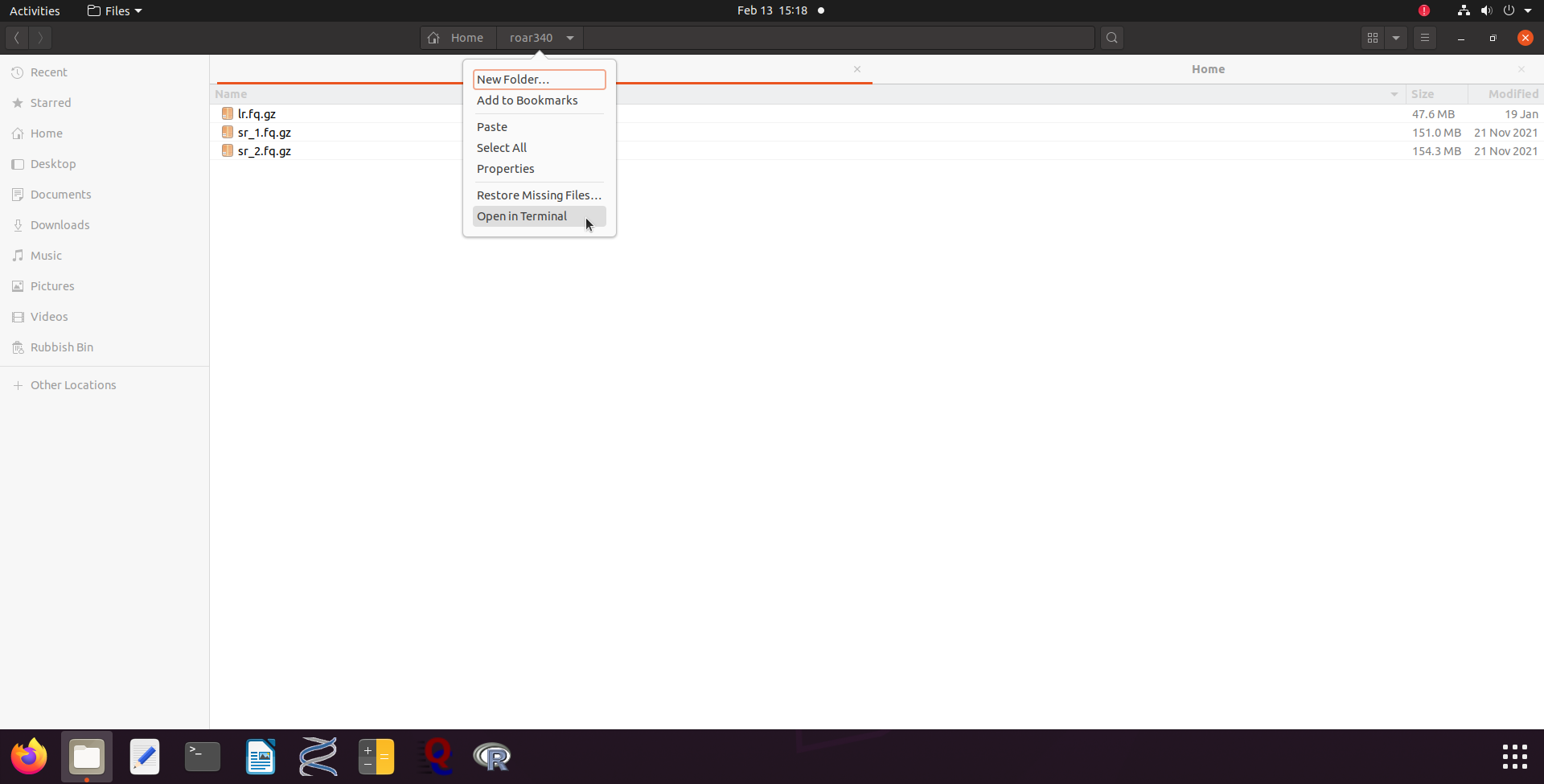


Unzip the sr_1.fq.gz file using this command:

$ gzip --decompress --keep sr_1.fq.gz

NOTE: the --keep option (also called a 'flag') signals to the gzip program to not delete the file we are unzipping

Next, we want to look at the fastq file to see what they look like. Because they are so big, if you used the cat command you would be faced with just scrolling letters and it could crash your computer.

Instead, we will just take a peak into the top and bottom of the file using the head command, which we used last week.

$ head -n 5 sr_1.fq

**Questions to answer in your notebook:**

1. Describe what the head command shows you.

2. Do the fastq file contents conform to the expected standard we learned in Lecture 4?

- 1. **Let’s confirm that our fastq file conforms to the standard of 4 lines per sequence.**

How could we do this? One way would be to count the number of lines in the file and see if it is divisible by 4. Using the wc command we learned last week (Pipes and Filters section) let’s count the number of lines in the sr_1.fq file.

$ wc -l sr_1.fq

NOTE: the -l option tells wc to count lines, not words.

**Questions to answer in your notebook:**

1. Is the number you got divisible by 4?

2. How many reads are in your sr_1.fq file?

1. **Examining your Illumina short read dataset using FastQC**

We got some information about the read files by inspecting them, but they contain hundreds of thousands to millions of reads, so we need some systematic way to examine them for problems and summarize that information. Luckily, there is a program for that.

The program **FastQC** is a program written in the java language that can help you examine the fastq files and show the quality of the sequence within them.

We will run this analysis on the zipped files now, leaving aside the unzipped (fastq) files for the time being. We do this because the zipped files take up less space and are just as efficient for the bioinformatic tools to access.

- 1. **Run FastQC on your short read _1 file.**

$ fastqc sr_1.fq.gz

• you should not see anything except for the words “Approx xx% complete for sr_1.fq.gz

• the analysis should take less than 5 mins

- - 1. **Learn Terminal shortcuts: arrow keys to access command history**

One way to avoid having to re-type all of the commands we are going through is to use the keyboard arrow keys.

To recall the command that was just used in Terminal, use the up arrow key on the keyboard. Continuing to click up will move further back in time while clicking the down arrow will move to newer commands.

Therefore, to run the same command as we just did but for the sr_2.fq.gz file, use the up arrow and then modify the text to replace the _1 with _2.

$ fastqc sr_2.fq.gz

- 1. **After fastqc is finished, look at the files created:**

$ ls

• You should see two new files created with the ‘.html’ file extension

sr_1_fastqc.html

sr_2_fastqc.html

- 1. **Open the two files created by fastqc using Firefox:**

NOTE: to save yourself time typing and to be more accurate, you should be trying to use 'tab completion' whenever possible. Recall from Software Carpentry lesson in Workshop 1: <https://swcarpentry.github.io/shell-novice/02-filedir/index.html#sorting-output>

$ firefox sr_1_fastqc.html

**Questions to answer in your notebook:**

1. What is the average sequence length of these reads?

2. What is the average GC% in these reads?

3. Describe how the quality scores vary across the read_1 and read_2 samples.

4. Are the read_1 reads good quality?

5. Are the read_2 reads good quality?

6. Are there any problems flagged with any of the FastQC categories? If so, what is the category and what does the error mean? Why might the error have occurred?

1. **Trimming poor quality sections from the Illumina reads**

Based on your FastQC analysis, it looks like at least some of your reads have regions of low quality and possibly some adapter sequences, either in the 5’ end and/or 3’ end. These low-quality regions should be trimmed off before we attempt to assemble the reads because they could result in misassembly or failed assembly.

Read trimming is the process of computationally shortening the reads by removal of bases of low quality. Additionally, read trimming can remove any adapter sequences that were sequenced due to short inserts. The sequencing adapters are short bits of sequence that are added to the end of DNA to be sequenced so that they can interact with the sequencing machines (see Lecture 4).

Use Trimmomatic to look for poor quality regions and leftover adaptors:

$ java -jar /usr/share/java/trimmomatic-0.39.jar PE sr_1.fq.gz sr_2.fq.gz sr_tr_1_paired.fq.gz sr_tr_1_unpaired.fq.gz sr_tr_2_paired.fq.gz sr_tr_2_unpaired.fq.gz ILLUMINACLIP:TruSeq2-PE.fa:2:30:10:2:keepBothReads LEADING:3 TRAILING:3 MINLEN:36

NOTE:

This trimming process has now stored your sequencing files with new names that you will use in the following steps:

sr_1.fq.gz The original forward (_1) reads

sr_tr_1_paired.fq.gz The reads that made it through trimming and so did their mate read

sr_tr_1_unpaired.fq.gz The reads that made it through trimming but their mate didn't so now are unpaired

sr_2.fq.gz The original reverse (_2) reads

sr_tr_2_paired.fq.gz The reads that made it through trimming and so did their mate read

sr_tr_2_unpaired.fq.gz The reads that made it through trimming but their mate didn't so now are unpaired

- 1. **Run FastQC on the trimmed reads.**

$ fastqc sr_tr_1_paired.fq.gz

and

$ fastqc sr_tr_2_paired.fq.gz

**Questions to answer in your notebook:**

1. Do the trimmed reads look different from the initial reads? If yes, how?

1. **Short read assembly using minia**

In this step we will perform an initial draft assembly of the short Illumina reads.

- 1. **Assemble the short reads using minia**

More info on minia: https://github.com/GATB/minia

- 1. First, we need to make a text file listing all the read files we want to assemble. This is needed as an input to minia. We will use several commands you have already seen last week including echo, pipes |, and > symbol to send the output to a file.

$ echo sr_tr_1_paired.fq.gz sr_tr_2_paired.fq.gz sr_tr_1_unpaired.fq.gz sr_tr_2_unpaired.fq.gz | xargs -n 1 > minia_input_reads.txt

- 1. **Let’s check that the minia-input-reads.txt file was created and see what is inside:**

$ cat minia_input_reads.txt

You should see this, showing that each input name was put onto its own line:

﻿sr_tr_1_paired.fq.gz

sr_tr_2_paired.fq.gz

sr_tr_1_unpaired.fq.gz

sr_tr_2_unpaired.fq.gz

- 1. **Now lets run minia to assemble the short Illumina reads:**

$ minia minia_input_reads.txt 31 3 4700000 minia

The code arguments in order of left to right:

minia runs the minia program

minia_input_reads.txt file showing which read files we want to assemble. NOTE: these need to be in the current directory.

31 the k-mer size

3 a k-mer will only be used in the assembly if it is observed this many times or more in the dataset. Used to remove low abundance k-mers that are likely erroneous.

4700000 the estimated size of the genome to be assembled (4,700,000 base- pairs). This is based on an average *E. coli* genome size.

minia the output file prefix

The assembly should take a few minutes

- 1. **Examine the output from minia**

At the end of the minia output is some important information on what the output of the assembly produced:

Extrapolating the number of branching kmers from the first 3M kmers: 4576

Looping through branching kmer n° 49800 / 49902 total nt 4909269

Total nt assembled 4909269 nbContig 698

Max contig len 106565 (debug: max len left 76268, max len right 49979)

Debug traversal stats: 1390 ends of contigs (577 unsaved small contigs), among them:

13 couldn't validate consensuses

0 large bubble breadth, 21 large bubble depth, 346 marked kmer, 25 no extension

566 in-branchin large depth, 317 in-branching large breadth, 98 in-branching other

-------------------Assembly time Wallclock 24.5328 s

-------------------Total time Wallclock 239.904 s

Although this is very complicated, by looking at only three pieces of this output we can learn a few things about the produced assembly:

Total nt assembled 4909269 This means that our sequence assembly included **4,909,269 nt** (or 4.9 megabases Mb) , which is a good number and is similar in size to a bacterial genome (e.g. *E. coli* has ~4.7 – 5.7 Mb genome)

nbContig 698 This means that the number (nb) of contigs assembled was **698**. This is a fairly large number since to have a complete genome assembly we are hoping for only having one contig.

Max contig len 106565 This means the largest assembled contig was **106,565 nt**. This is pretty good, but since we expect a complete bacterial genome to be ~4.7 – 5.7 Mb, and this largest contig is only 0.1 Mb, we still have a way to go before having a complete assembly.

- 1. **Examine the assembly files**

After the assembly is complete you can see what new files were created by using the ls command.

- 1. **But the results of the ls command are a bit too complicated. You can always pipe (|) the results of commands through the grep command to simplify. For example:**

$ ls | grep minia

• this code sends the results of the ls command through grep to filter out only those results that match the words we’ve given it (minia). This greatly reduces the complexity of what you have to look at. You can do this for any ls commands throughout the rest of the workshop if all the files are getting too complicated to look at.

- - 1. **Now let’s take a look at the contig file containing the assembled reads. Using the cat command, let’s look at minia.contigs.fa**

$ cat minia.contigs.fa

**Questions to answer in your notebook:**

• What do you see when you run this command?

- - 1. **That’s a bit too much information all at once! What if we used the head or tail commands instead to see the very top and bottom of the file?**

$ head -n 2 minia.contigs.fa

﻿

$ tail -n 2 minia.contigs.fa

﻿

**If we just wanted to see the FASTA definition line for each contig in our file, we could use cat, grep, and pipes in one combined command.**

$ ﻿cat minia.contigs.fa | grep ">"

Now let's see how many contigs there are using some of the same commands we used before piped together:

$ cat minia.contigs.fa | grep ">" | wc -l

It’s a bit hard to get into from this about how good the assembly was, although the presence of >400 contigs means that we are a long way from having a final assembly with one continuous chromosomal assembly.

We could continue looking at the contig data for a while to get more info, but luckily there is software that can do this more quickly and systematically than we can!

1. **Check assembly quality with QUAST**

QUAST stands for **QU**ality **AS**sessment **T**ool. The tool evaluates genome assemblies by computing metrics about the quality of the assembly.

- 1. **Let’s first run QUAST on our minia assembly without a reference sequence. This limits the information QUAST can return on our assembly but gives us a place to start.**

$ python /home/genomics/miniconda2/bin/quast -o quast_minia/ minia.contigs.fa

- remember to keep your data organised and record where the files for each analysis are stored. It will get confusing very quickly!

Quast has output the files from its analysis in the folder quast_minia/.

To find out where you are currently in the folder structure type:

$ pwd

Navigate into the new directory created to hold the QUAST output and look at the files.

- - 1. **You can look at the report to see what QUAST has produced using Firefox:**

Once inside the folder containing the results of QUAST analysis of the minia assembly:

$ firefox icarus.html

**Questions to answer in your notebook:**

• What is the N50 for your minia assembly?

• What is the L50 for your minia assembly?

• How do these values compare to what you saw by looking at the raw output of minia (section 6.5)?

1. **Improving the minia assembly**

There were several parameters that we set when we originally ran mina.

$ minia minia_input_reads.txt 31 3 4700000 minia

Could we improve the minia assembly by varying these parameters? Let’s see!

**General form of the minia command:**

minia <input_file> <kmer_size> <min_abundance> <estimated_genome_size> <prefix>

Run minia again and varying the ‘min_abundance’ parameter from 3 to 15. We will also vary the prefix parameter so that the results are stored in a different folder from our previous results.

$ minia minia_input_reads.txt 31 15 4700000 minia_15

- 1. **Examine the output from minia**

As before in section XX above, look at the following three outputs to see how they compare.

**Questions to answer in your notebook:**

• How does changing the ‘min_abundance’ parameter from 3 to 15 change the outputs from minia?

Total nt assembled

nbContig

Max contig len

• Why might the sequence assembly change in this way when we chose to only use kmers that appeared 15 times or more in the sequence data?

- 1. **Increasing the ‘min_abundance’ parameter further.**

Since increasing the kmer ‘min_abundance’ from 3 to 15 improved the assembly, let’s try to improve it further by running minia again with ‘min_abundance’ set to 20.

$ minia minia_input_reads.txt 31 20 4700000 minia_20

**Questions to answer in your notebook:**

• How does changing the ‘min_abundance’ parameter from 3 to 20 change the outputs from minia?

Total nt assembled

nbContig

Max contig len

• Why might the sequence assembly change in this way when we chose to only use kmers that appeared 20 times or more in the sequence data?

**That is the end of our short read only analysis.**

1. **Long Read (Nanopore) Genome Assembly using Raven**

Third-generation sequencing technologies provided by Pacific Biosciences and Oxford Nanopore Technologies generate read lengths in the scale of kilobasepairs. However, these reads display high error rates, and correction steps are necessary to realize their great potential in genomics.

We will use an assembly method made specifically for long read (3^rd^ generation) technology.

More info about Raven: <https://github.com/lbcb-sci/raven>

- 1. **First, make a new directory (called /roar340/raven/) to hold all of the assembly files:**

Return to the main roar340/ folder. If you are currently in the quast_minia/ folder then you must move back one level in the directory structure:

$ cd ..

**Once pwd command shows you are in the roar340/ folder:**

$ mkdir raven/

- 1. **Run Raven to assemble the long reads:**

$ raven -t 3 lr.fq.gz > raven/raven.fa

- 1. **Next, run QUAST to evaluate assembly quality as done previously.**

$ python /home/genomics/miniconda2/bin/quast -o quast_raven raven/raven.fa

Where:

• The Quast output folder is called: quast_raven

- 1. **Examine your QUAST results for Raven and compare to your QUAST minia results.**

**Questions to answer in your notebook:**

• What is the N50 for your long read assembly?

• What is the L50 for your long read assembly?

• Is the long read assembly (Raven) better or worse than the short read (minia) assembly? What factors did you consider when coming to this conclusion?

***** You have almost completed Workshop 2. Congratulations! *****

***** Shut down your virtual machine in the same way you would shut down a computer *****

1. Click the down triangle in the top right corner of the virtual machine desktop.

2. This will bring down a menu and you will **click ‘’Power Off/ Log Out**” which opens another menu

3. In the second menu **click “Power Off”** which will bring up another window where you either wait 60 seconds and it will power off itself or you **click “Power Off” again**.

**
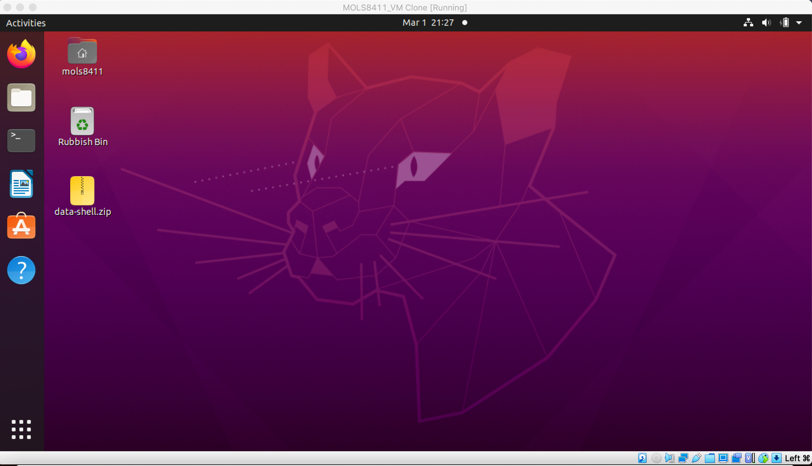
**

**
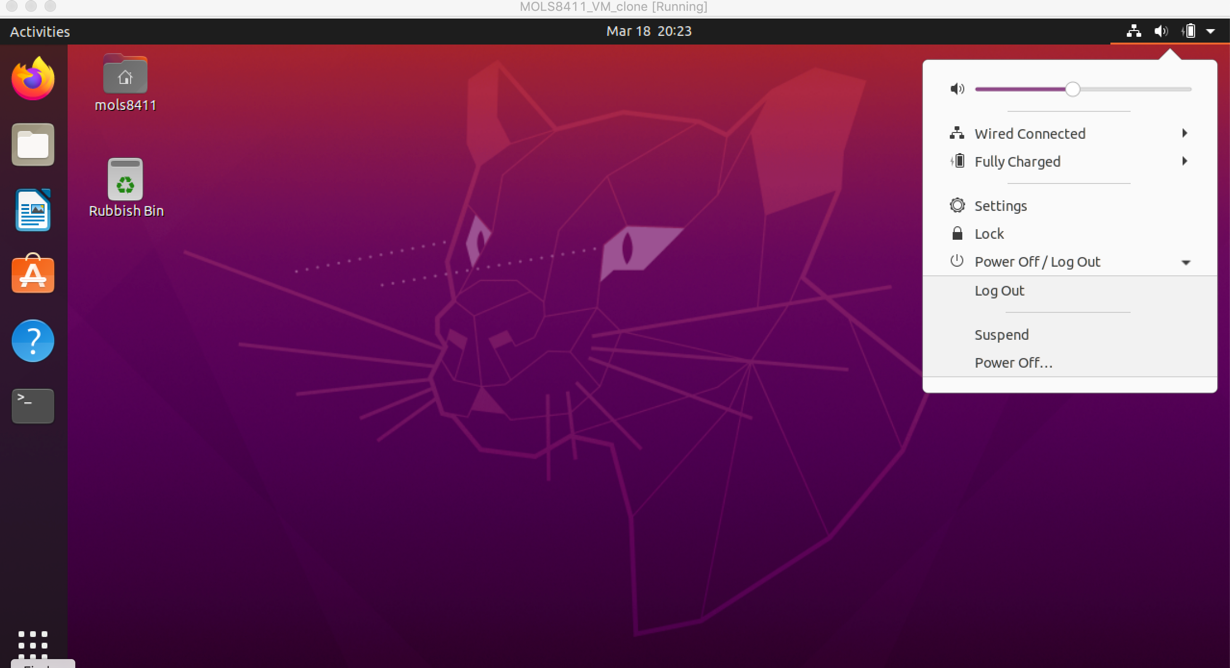
**

**
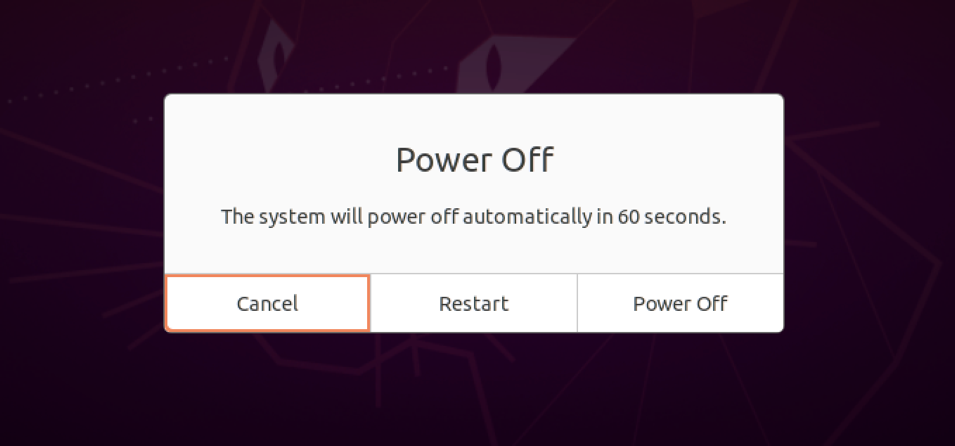
**

**WARNING**: make sure you shut down the virtual machine properly when you are done for the day or all your data from today may be lost and your grade may be adversely affected!
