# Supplementary material for: A bacterial genome assembly and annotation laboratory using a virtual machine
Source: Biochem Mol Biol Educ. 2023 Mar 3;51(3):276–85. doi: 10.1002/bmb.21720 (PMC10947226; doi:10.1002/bmb.21720)
Supplement: Supplementary file 3 — Data S3 ‐ Hybrid Assembly [file BMB-51-276-s001.docx]

Workshop 3: Hybrid sequence assembly using SPAdes, and genome annotation

Last week we learned how to use the Bash Shell and used it to quality check the sequencing reads and to assemble these reads into contigs and scaffolds. Although we were able to assemble the reads we were not able to successfully create a full assembly with no misassemblies for the entire genome.

Today we will use a tool that combines the advantages of short reads (high coverage) and long reads (high contiguity) while compensating for the disadvantages of short reads (poor contiguity) and long reads (poor coverage). Following the assembly we will map reads back to the assembly to check how well the assembly performed. Lastly, we will use a tool to find genes on our sequence and complete the annotation of the genome.

Today you will be working in your normal groups of two.

**Reminder: Record Keeping**

Keep track of your work in a notebook like OneNote or in a Word file. This lets you record your workflow logic as well as the code you used to get your results. **Your virtual machine could crash and lose all your data** but if you keep track of the steps and commands you execute you can reconstruct what you’ve done and quickly recover. **You will hand in your notes as part of the Workshop Assessment**.

1. **Start your Virtual Machine**

Today, we will be doing all the analysis from inside the virtual machine. Start it up using the same method as is outlined in Workshop 1.

**WARNING**: make sure you shut down the virtual machine properly when you are done for the day. See Workshop 1 student notes for the protocol.

1. **Hybrid assembly with SPAdes**

• Navigate to the directory containing your Illumina and Nanopore reads (roar340/) and execute this code:

$ spades -t 3 -m 18 -1 sr_tr_1_paired.fq.gz -2 sr_tr_2_paired.fq.gz --nanopore lr.fq.gz -o spades/

• NOTE: It may take up to 30 mins to run this code.

The results will be a file located in the spades/ folder called: scaffolds.fasta

- 1. **Next, run QUAST to evaluate assembly quality as done previously.**

While in the roar340/ folder, execute this code:

$ python /home/genomics/miniconda2/bin/quast -o quast_spades/ spades/scaffolds.fasta

- - 1. **Look at the report to see what QUAST has produced:**

$ firefox quast_spades/icarus.html

- - 1. **While looking at the icarus.html file also click on the words in the top left ‘View in Icarus contig browser’.**

**In your notebook:**

• What is the N50 for the SPAdes assembly?

• What is the L50 for the SPAdes assembly?

• Describe how the SPAdes hybrid short plus long hybrid assembly is different than the short read assembly and long read assemblies alone.

1. **Comparing the short-read only (minia), long-read only (Raven), and hybrid assembly (SPAdes)**

We can see through the L50 and N50 data that the SPAdes hybrid assembly is better than the short or long-read only assemblies but it would be a good idea to see how they compare directly.

- 1. **Create a new folder to house our Quast analysis:**

From roar340/ folder:

**(1) From the CLI:**

$ mkdir quast_hybrid/

**(2) Alternatively, open the Files GUI**

make a folder using the hamburger icon (three horizontal lines) on the far right of the page, followed by selecting the folder icon on the far right of the flyout menu. Name it quast_hybrid


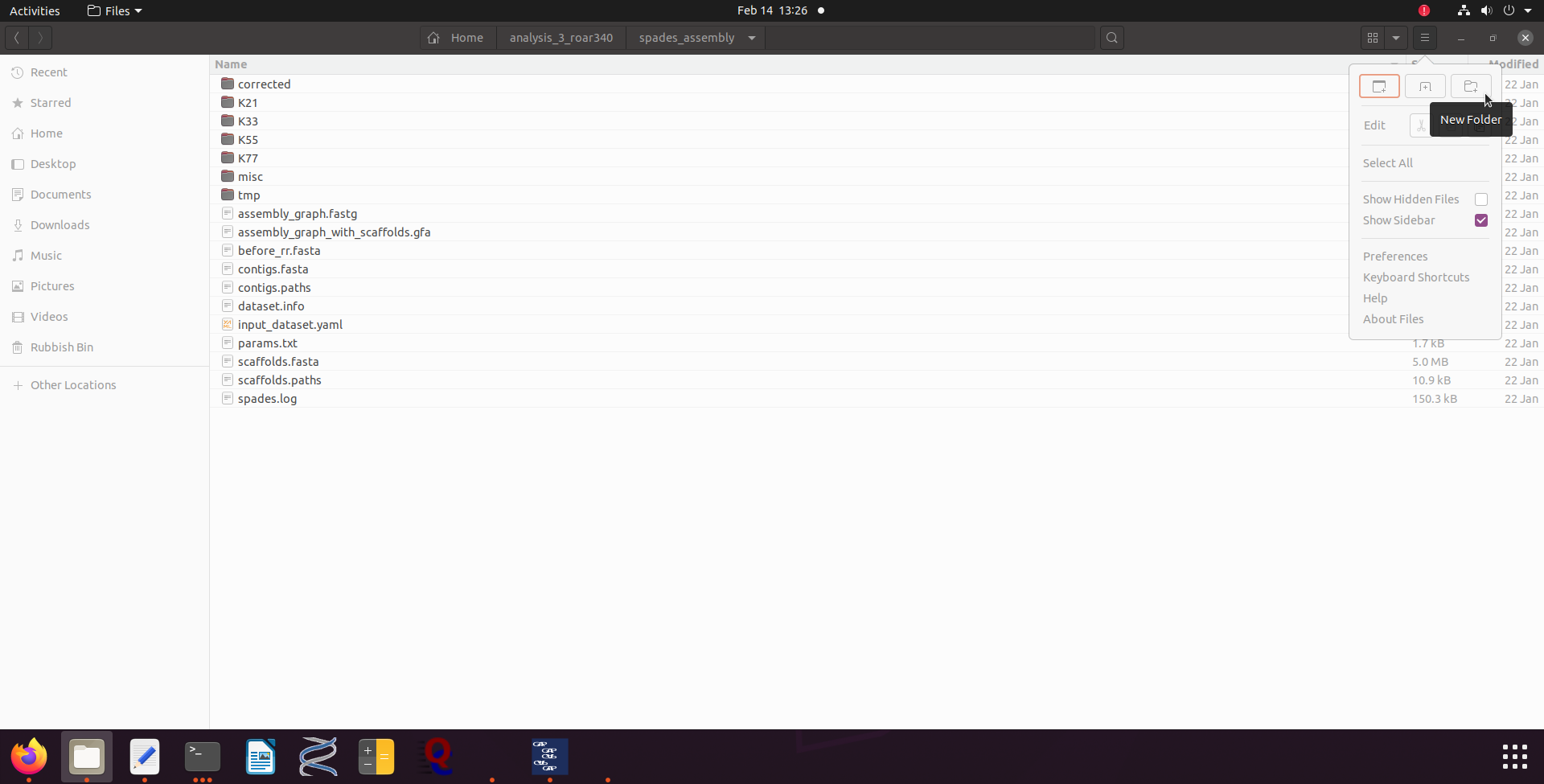


- 1. **Locate assembly files:**

**minia assembly:**

**• Depends on which sample you're using:**

roar340/minia.contigs.fa

**Raven assembly:**

raven/raven.fa

**SPAdes assembly:**

spades/scaffolds.fasta

- 1. **Copy the minia, Raven, SPAdes assembly files into the newly created folder (quast_hybrid/)**

You can use either Files or CLI to do this. When you're done the folder should look like this.


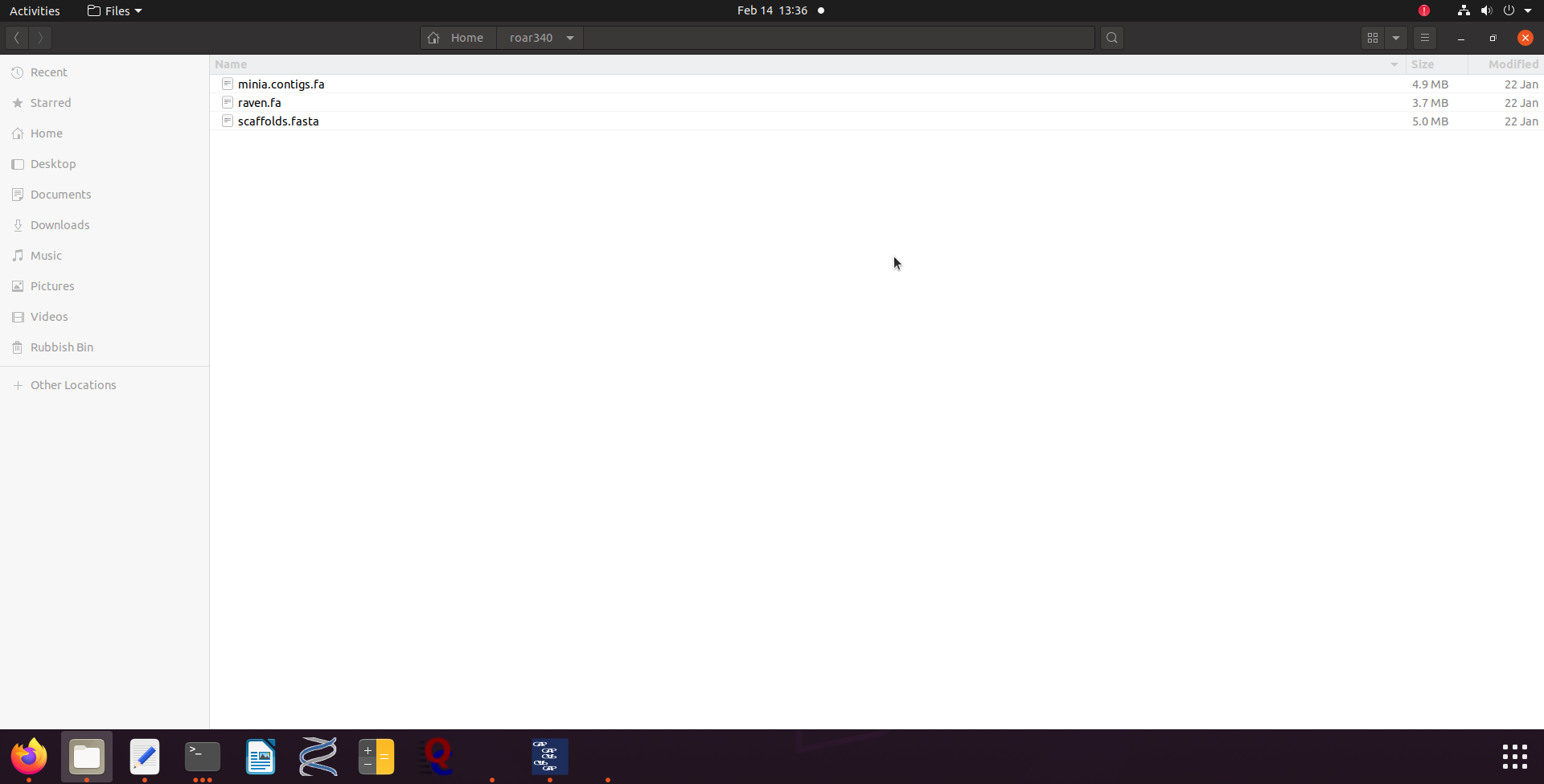


$ ls

minia.contigs.fa raven.fa scaffolds.fasta

And if you invoke pwd using Terminal you should get this:

$ pwd

/home/genomics/roar340/quast_hybrid

- 1. **Run QUAST from this directory using CLI**

We want to align the minia and Raven contigs/scaffolds to the SPAdes hybrid assembly so we treat the SPAdes assembly as a reference and use QUAST to perform the alignment and create statistics of how well the short and long-read only contigs map across the larger SPAdes scaffold.

$ quast minia.contigs.fa raven.fa -R scaffolds.fasta -o quast_results/

- 1. **Explore the Quast results**

Navigate into the newly created sub-folder quast_results/

Either using the CLI or Files GUI, select and run the icarus.html file.

**CLI:**

$ firefox icarus.html

**Files:**


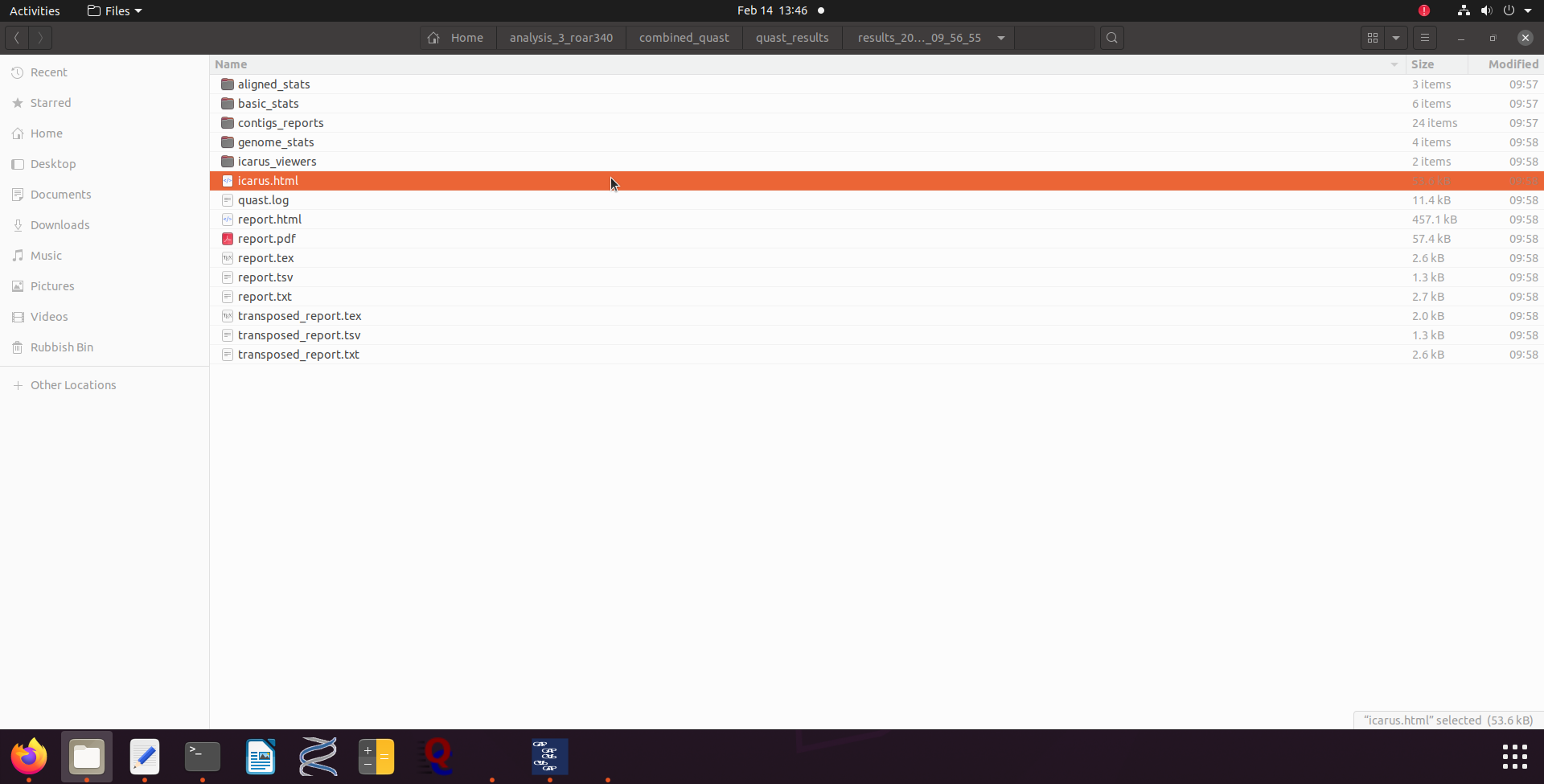


- 1. **Contig alignment viewer**

Select Contig alignment viewer from the main Icarus page. This will take you to a graphical view of where the minia and Raven contigs aligned to the SPAdes assembly. On the top is a detailed view that can be zoomed in and out using buttons on top of screen. In the middle of the screen is a large-scale view of the alignment. You can move the highlight box to different areas of the assembly to investigate in more detail in the above detailed pane.


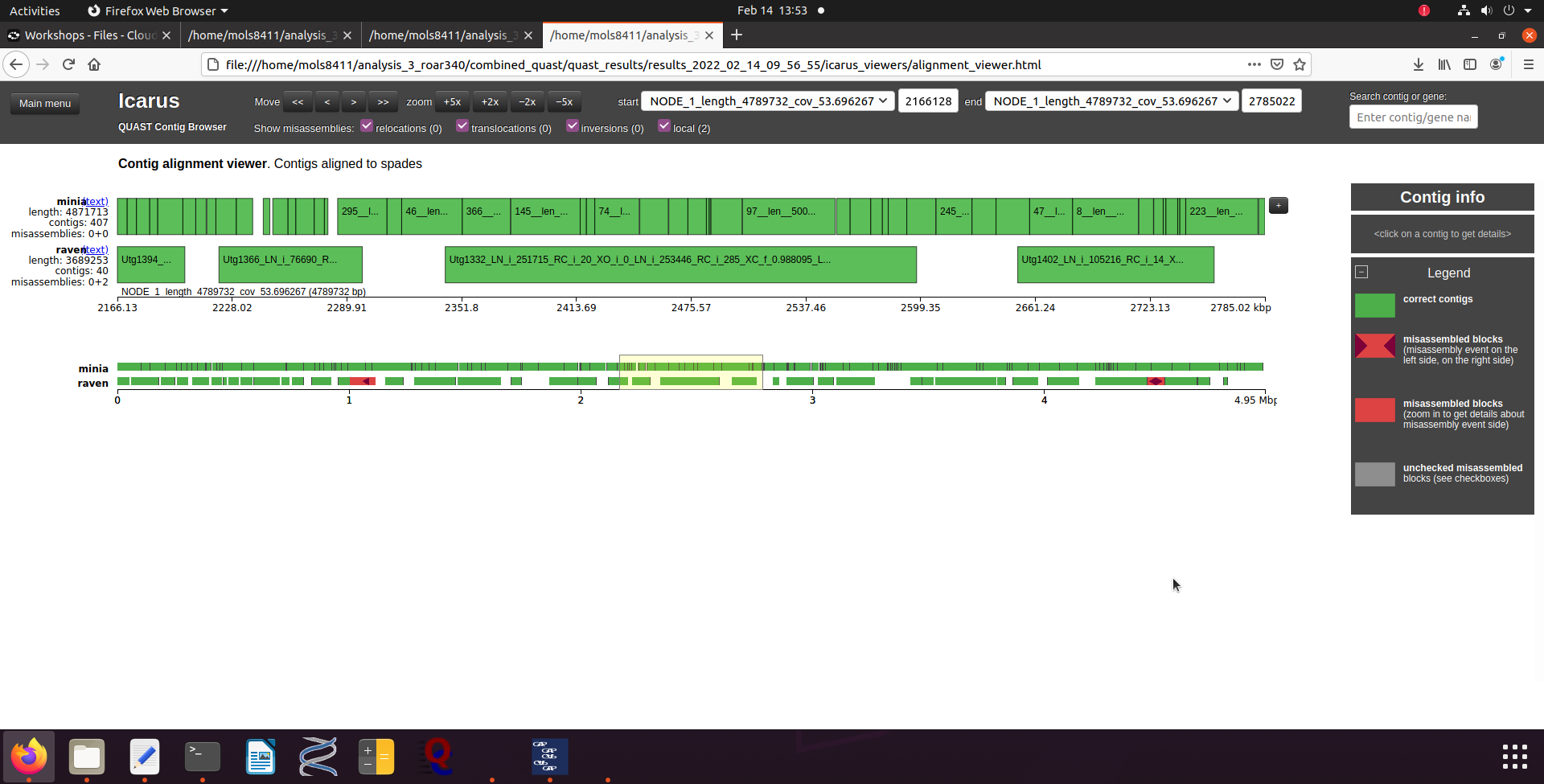


**In your notebook:**

1. Do you see any misassembled blocks? Which type of assembly created these?

2. Why might these have occurred?

- 1. **QUAST report**

Go back to the Main menu by clicking the button in the top left of the Icarus viewer. Now click on the words 'QUAST report'.

This report is different from the others you have seen before because it compares the minia and raven contigs to the SPAdes scaffolds.

**In your notebook:**

1. How many contigs (fragments) did SPAdes assembly result in? How many for minia and Raven?

2. Which assembly method (minia or Raven) performed the best based on the parameters displayed?

3. If you could only use the minia or Raven method to assemble a genome, which would you use? Why?

4. Which assembly method has a better N50? (hint: click on 'Extended report' to open up N50 info)

5. Which assembly method has a better NGA50?

6. What is the difference between N50 and NGA50? (hint: hover cursor over terms to get definitions)

7. Why didn't we see NGA50 values when we performed QUAST analysis on minia and Raven assemblies previously?

1. **Mapping reads back to the assembly**

To get visual feedback on how good the SPAdes hybrid assembly is, we are going to take the scaffolds.fasta file that was generated by SPAdes and use this as a reference sequence to separately map the Illumina and Nanopore reads back onto it. This will allow us to see where there are gaps or ambiguities and to visually determine if the assembly is ‘complete’ or if there are areas that could be improved manually.

- 1. **Using minimap2 to map short reads back to the SPAdes assembly.**

The first step involves generating a read map for coverage assessment using minimap2 tool and then sending that information directly to another tool called samtools. The output is a .bam file which is a binary version of a .sam file. A .sam file is a tab-delimited text file that contains sequence alignment data (discussed in lecture before Workshop).

**Info on minimap2**: https://lh3.github.io/minimap2/minimap2.html

**Info on samtools:** http://www.htslib.org/doc/samtools.html

Below, the first command aligns the short reads to the SPAdes scaffolds.fasta file.

Note that we are using the | command, or *piping* (workshop 1) the output of minimap2 to another tool called samtools to create a sorted bam alignment file. Piping cuts down on intermediate files because the results of one tool are not saved but passed directly through and increases computational efficiency.

$ minimap2 -ax sr spades/scaffolds.fasta sr_tr_1_paired.fq.gz -2 sr_tr_2_paired.fq.gz | samtools view -b | samtools sort -o sr.bam

• The following code will output a .bam file called sr.bam that you will use in a further step

- 1. **Map long reads to SPAdes assembly**

$ minimap2 -a -x map-ont spades/scaffolds.fasta lr.fq.gz | samtools view -b | samtools sort -o lr.bam

• The code will output a .bam file called lr.bam that you will use in a further step

**Questions to answer in your notebook:**

1. Examine the code and see if you can determine what each part (commands and options) does and then describe what is happening inside the above code in your notebook. Look up the ***view*** and ***sort*** commands in the samtools manual, the *options/flags* used, and look back to Workshop 1 to see what *piping* does.

**Hint, look here:**

**minimap2:** https://lh3.github.io/minimap2/minimap2.html

**samtools view:** http://www.htslib.org/doc/samtools-view.html

**samtools sort:** http://www.htslib.org/doc/samtools-sort.html

- 1. **Combine .bam files using samtools.**

We next want to create a combined .bam file containing both the short and long reads so we can examine them together in the same window. We can do this by invoking the merge command of the samtools package:

$ samtools merge combined.bam sr.bam lr.bam

- 1. **Next, the bam file has to be indexed using samtools.**

Indexing a sorted .bam file is needed to quickly extract alignments overlapping particular genomic regions. Indexing is also required by genome viewers to be able to quickly display alignments.

• The following code will create an index file (.bai) for the combined.bam file

$ samtools index combined.bam

1. **Visualising the mapped reads using gap5 in the Staden package.**

The gap5 program enables us to see the reads in a graphical visualisation as they relate to the contig generated by SPAdes assembly. If the reads look like they map across the entire sequence and there are few areas where low read density or gaps exist (deletions) or extra reads are piled up (genome duplications) then we can be confident to go on with the next step of the analysis.

**Visualisation of the bam file with gap5 and the Staden package**

gap5: https://www.sanger.ac.uk/tool/gap5/

Staden package: http://staden.sourceforge.net/

Before the gap5 program can be used to look at the reads we need to generate another index file for the reads using the tg_index command.

$ staden tg_index combined.bam

• the gap5 tool created two files for the combined.bam file:

﻿sr.0.g5d

sr.0.g5x

- 1. **Open the sr.0.g5d file using gap5.**

$ gap5 combined.0.g5d

Gap5 is program with a GUI and can be used with both the mouse and keyboard.

The opening screen should look something like this:
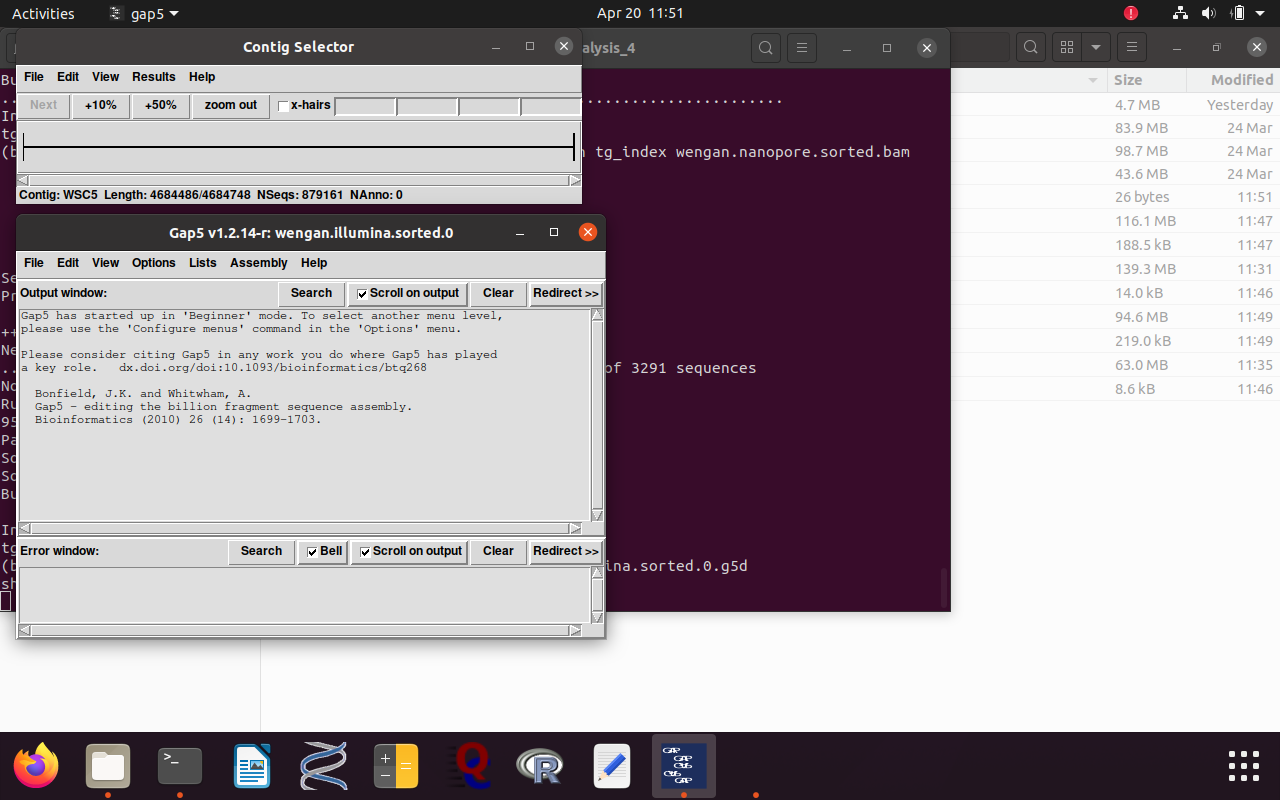


Next, put your mouse cursor over the contig representation (the black bar in the Contig Selector window). It should change pink when your cursor is on it. If there is more than one contig, select the largest one (usually left-most):


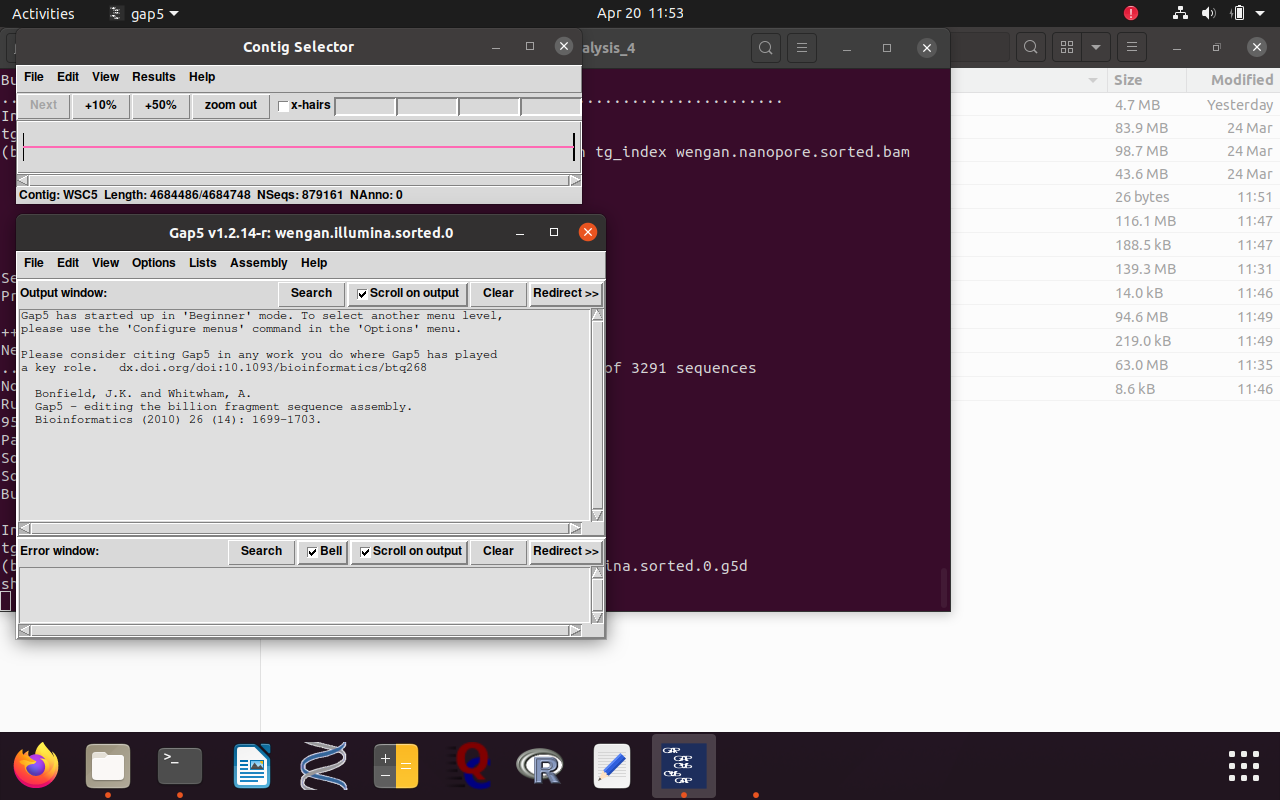


Next, right click on the contig and a menu should pop up. Select **Template display**:


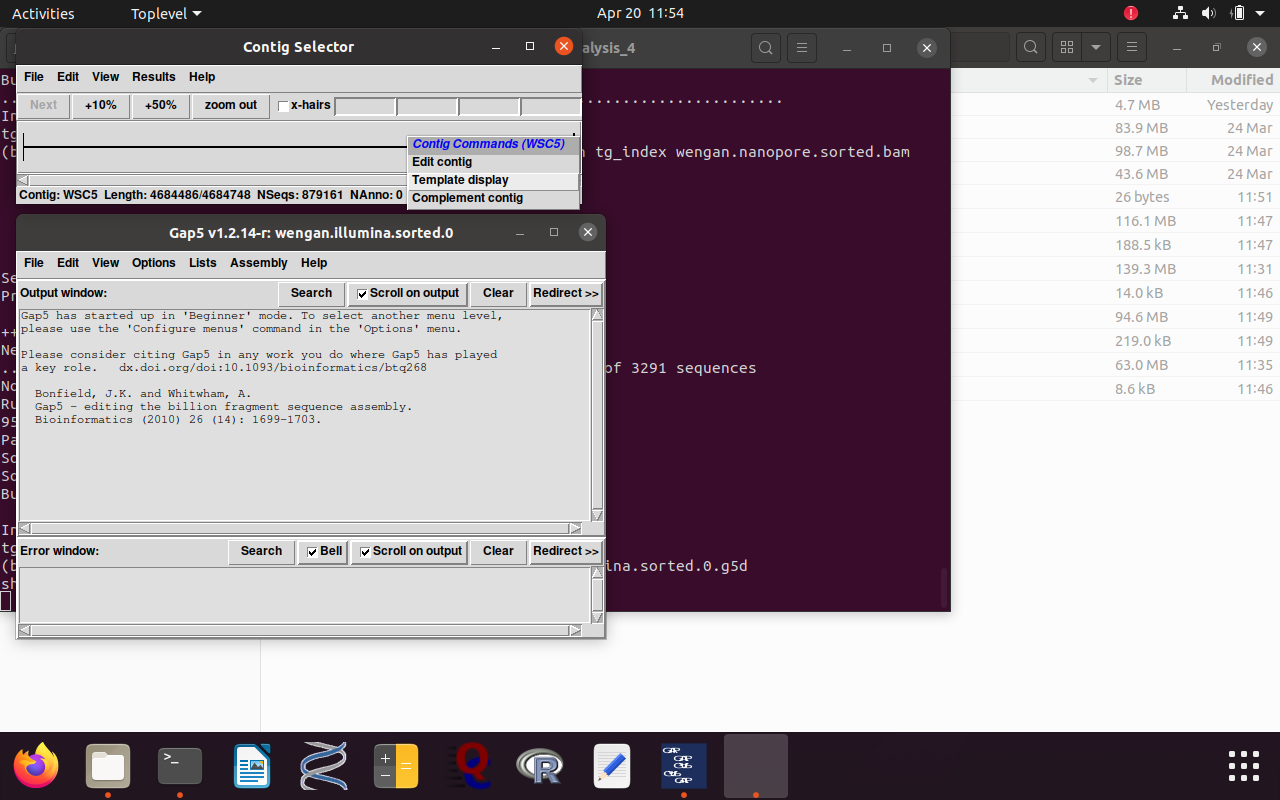


This will bring up the Contig window which shows you the contig line and an upper frame shows the reads in their position and extension relative to the contig. The colours of the reads have meaning explained below. In the lower frame shows a graph summarizing the coverage and the quality of the assembly (sequence / read-pair coverage).


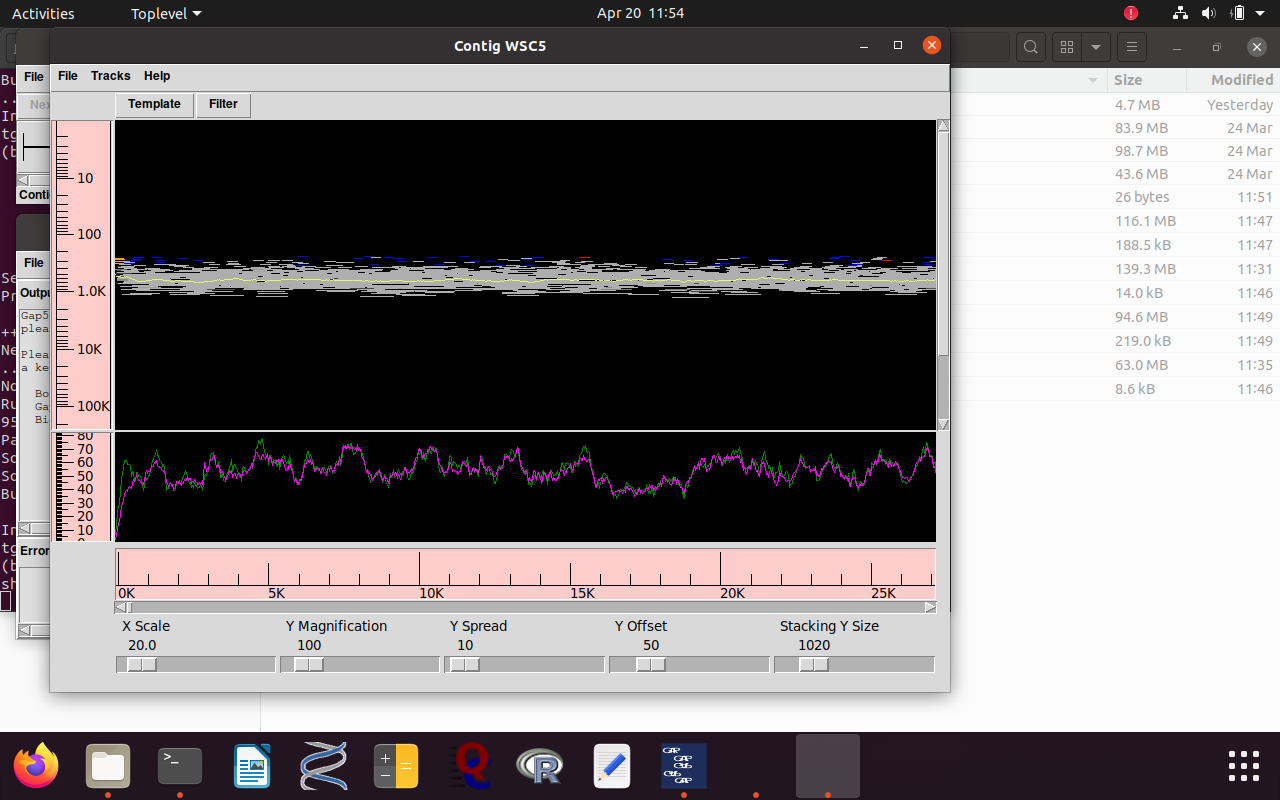


Below the main menu bar is a series of buttons that bring up new dialogues for controlling how the data is to be display and what is to be displayed.

You can zoom in and out and move side to side across the contig using the mouse and slider bars.

The X-scale simply controls how many base-pairs of the contig are covered by the window. The X-scale number is arbitrary, but is interpreted in an exponential manner so it is easy to rapidly zoom in or zoom out.

**What do the colours mean?**

**Blue** This is a template with only one reading present. It could be either a pair with one end not in this assembly, or a true single-ended sequencing experiment.

**Orange** This is a template with one read present in another contig. The size of the line is derived from the size of the data in this contig (typically a single reading).

**Red** This template is considered as inconsistent in some manner, typically due to the relative position and orientation of the forward and reverse sequences being incorrect.

**Grey** Any consistent read-pair is coloured by the mapping quality, by default using the average of the individual sequence mapping qualities. Lighter shades represent higher mapping qualities.

- 1. **Zoom out and explore the contig and both the short and long reads that map to it.**

**Questions to answer in your notebook:**

1. Are there any regions with only short reads mapping to it?

2. Are there any regions with only long reads mapping to it?

**OK! The assembly looks good enough to continue on to the annotation step.**

1. **Prokka annotation**

Now that we’re fairly confident that our assembly was high quality and we have the genus of we can proceed to annotate the genome. We will be using one of the more popular programs called Prokka to annotate the genome. Prokka combines several existing tools to perform *ab initio* gene finding followed by homology searching to confirm these initial provisional genes. Additionally, homology search across several gold standard databases is used to add functional annotations onto the structural annotation information.

Reference: Torsten Seemann, Prokka: rapid prokaryotic genome annotation, Bioinformatics, Volume 30, Issue 14, 15 July 2014, Pages 2068–2069, https://doi.org/10.1093/bioinformatics/btu153

- 1. **Run Prokka on the contig file created from SPAdes**

From the roar340/ folder:

$ prokka --prefix roar340 --locustag roar340 --kingdom Bacteria --genus Escherichia --usegenus --rfam spades/scaffolds.fasta

• NOTE: It may take up to 30 mins to run this code.

This code will produce a new directory called roar340/ containing the following files:


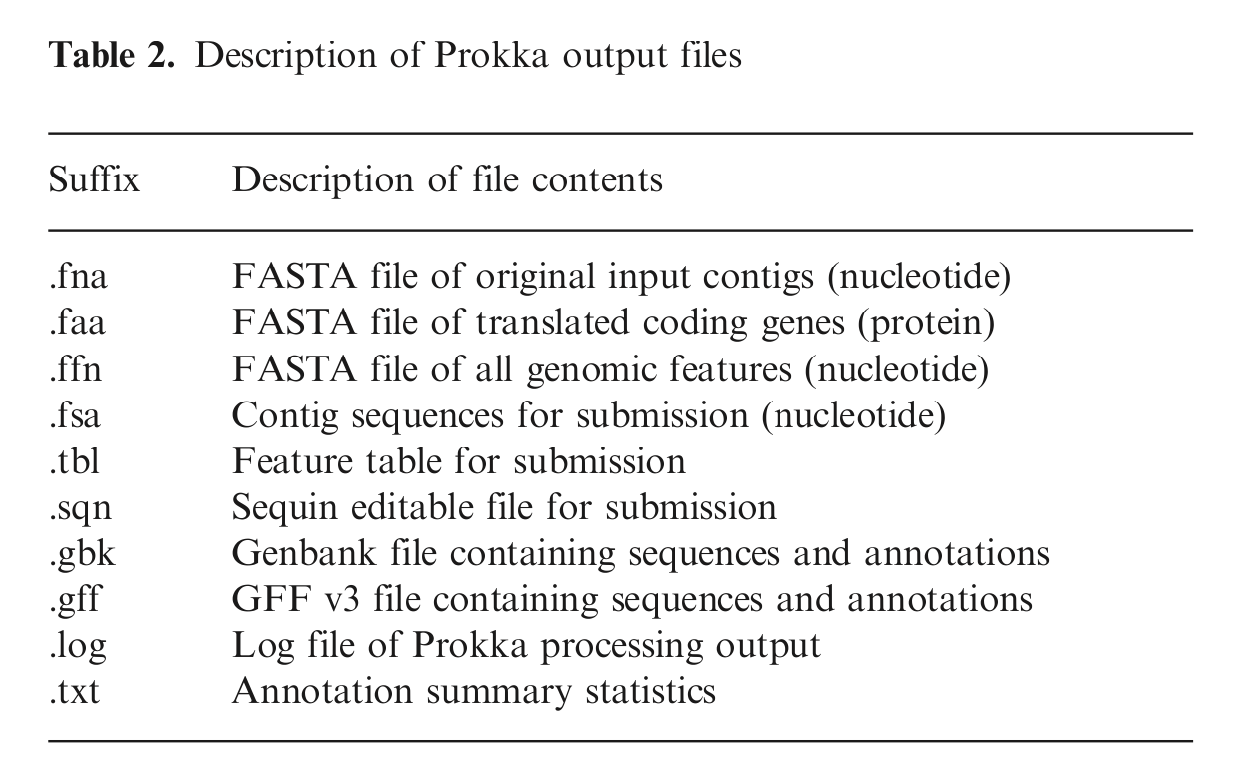


Torsten Seemann, Prokka: rapid prokaryotic genome annotation, Bioinformatics, Volume 30, Issue 14, 15 July 2014, Pages 2068–2069, https://doi.org/10.1093/bioinformatics/btu153

1. **Examine annotated genome with Artemis**

Artemis is a graphical user interface (GUI) genome browser. Unlike the genome browsers you may have used before, Artemis is not web-based and will only display information for your genome that was created by Prokka.

- 1. **Open Artemis by typing:**

$ art

**This brings you to the welcome screen of the program:**


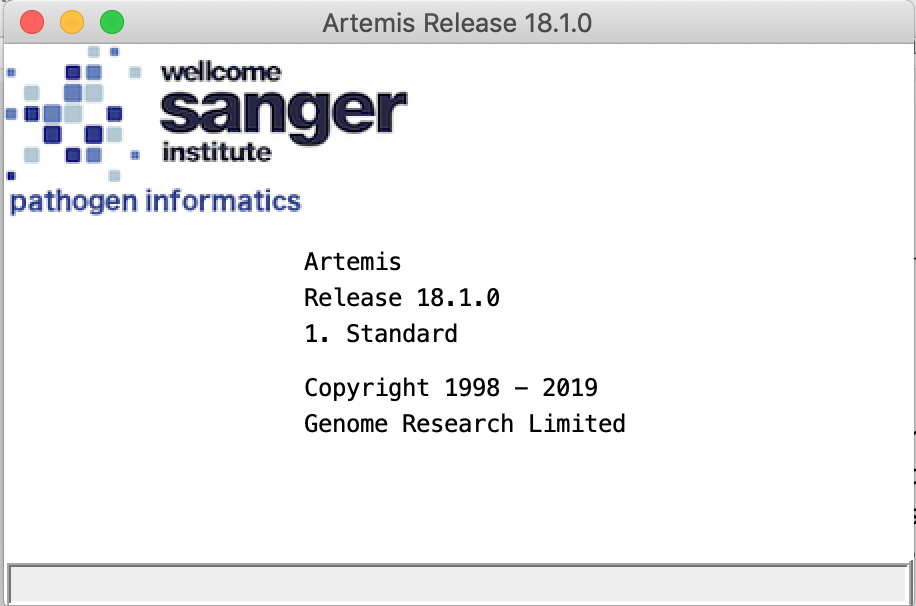


- 1. **From here click on File->Open and then locate your annotated genome GenBank file (roar340.gff) and click on it.**

**
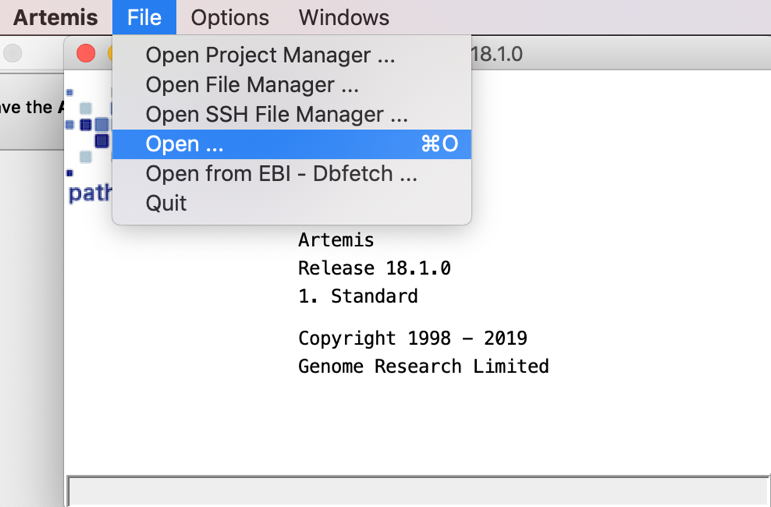
**

**
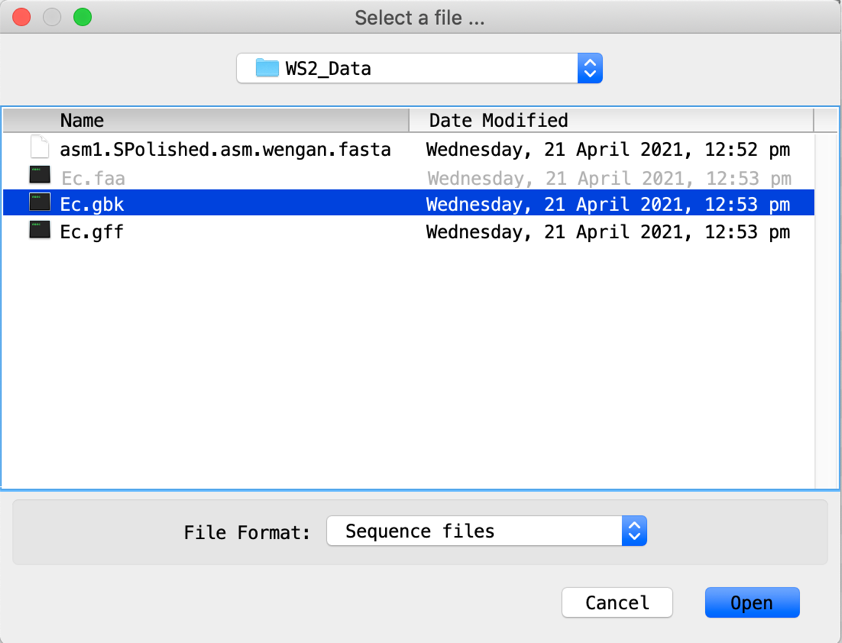
**

**
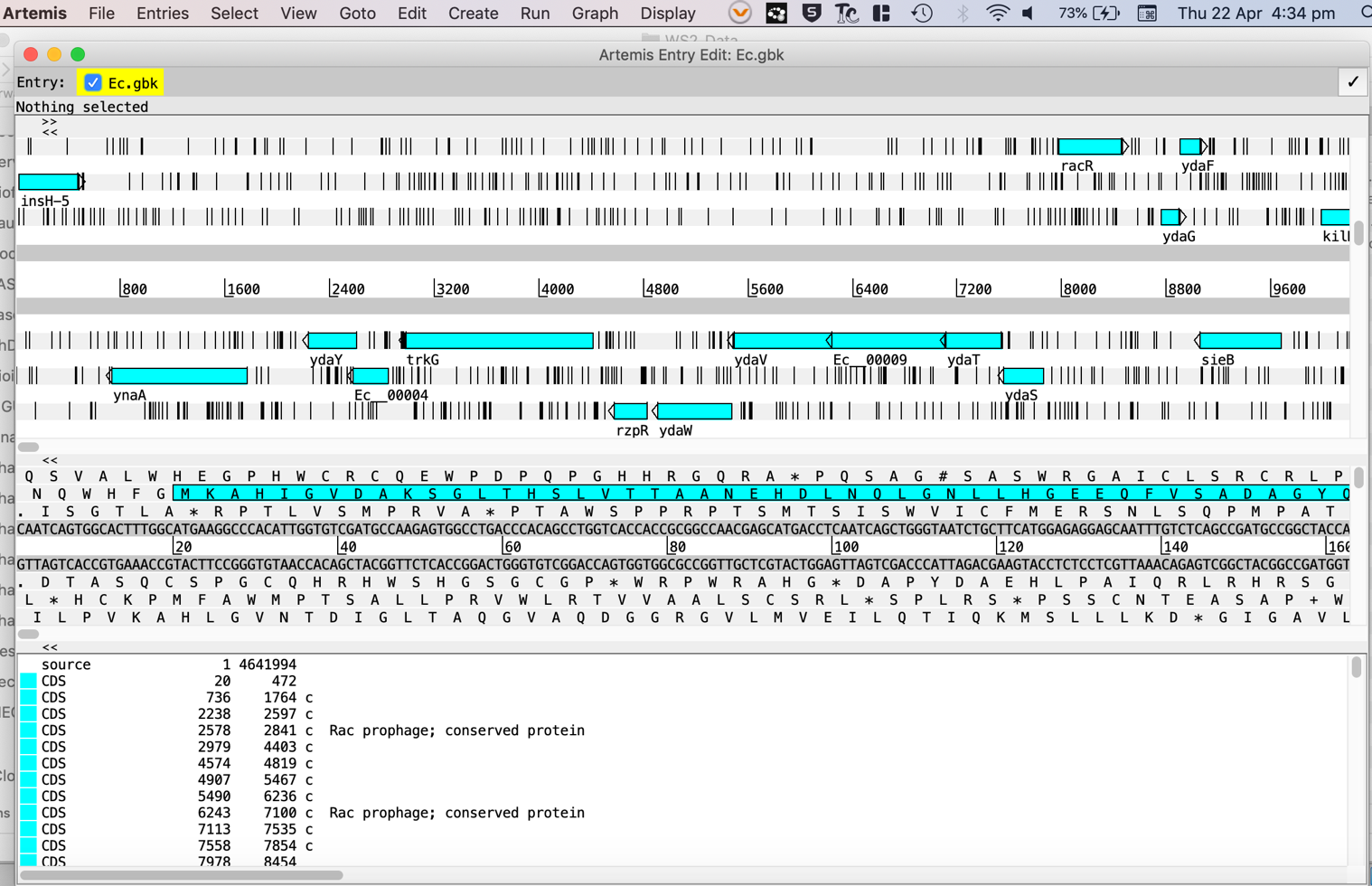
**

- 1. **Explore the sequence you just assembled from raw sequence reads into a fully annotated genome.**

The Artemis screen has three panes. The top pane has a gene-level large scale view of the genome. The three forward reading frames are on the top part of this frame with the numbered coordinates between the top Forward three frames going left to right, and below this the bottom Reverse three reading frames going left to right.

You can scroll left and right using the scroll bar at the bottom of the top pane.

The middle pane is a nucleotide and amino acid level view of what is selected in the top pane. You can scroll left and right using the scroll bar at the bottom of the middle pane.

The bottom pane is gives annotation feature information for whatever is selected in the top pane.

- 1. **Jumping to Features using the Navigator**

You can move quickly to features by clicking Goto->Navigator… which brings up the Artemis Navigator window. This window enables you to search using different criteria such as Feature with Gene Name and Base Pattern.


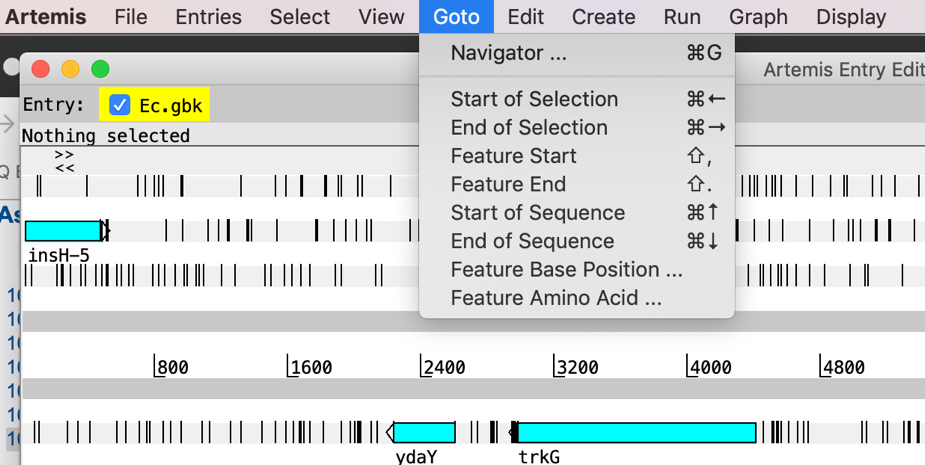


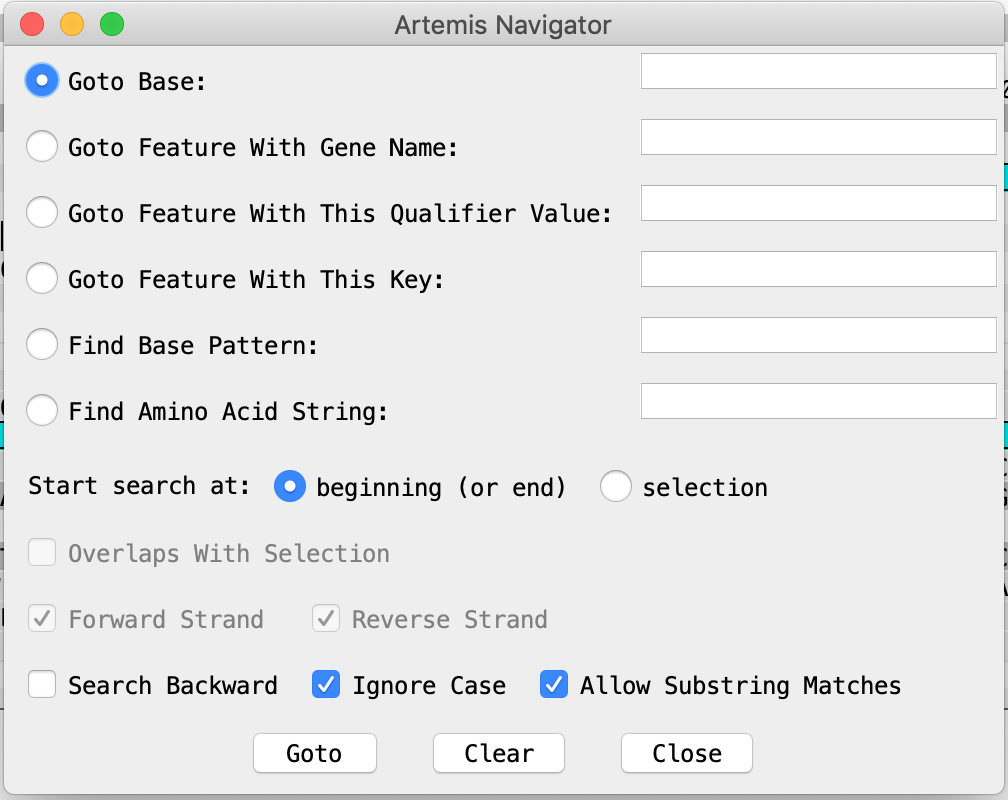


- - 1. **Find a 16S ribosomal RNA gene (hint: use Feature With This Qualifier Value)**
    2. **Find 3 different tRNAs and write down their coordinates.**
    3. **Find the name of the gene that encodes the L-fuculokinase enzyme (hint: use Goto Feature with this Qualifier Value).**
    4. **Take a global image of your genome using the File->Open in DNAPlotter command.**

**
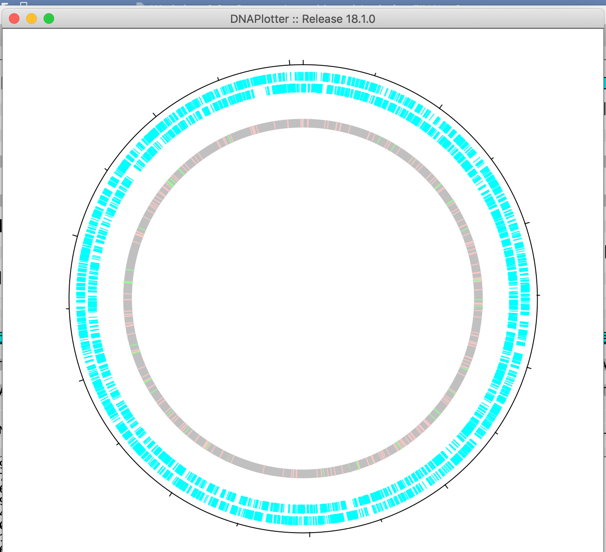
**

You can overlay info onto this standard view by using Options->Features to select a gene to display or select a region (start to stop coordinates) to highlight.

**And thus ends Workshop 3….you did it!**

**Next week we will use several tools to analyse the genome further and enable us to determine if ROAR340 is pathogenic or antibiotic/phage resistant?**
