# Supplementary material for: A bacterial genome assembly and annotation laboratory using a virtual machine
Source: Biochem Mol Biol Educ. 2023 Mar 3;51(3):276–85. doi: 10.1002/bmb.21720 (PMC10947226; doi:10.1002/bmb.21720)
Supplement: Supplementary file 4 — Data S4 ‐ Specialized Analysis [file BMB-51-276-s002.docx]

Workshop 4: Specialized sequence analysis and poster creation

Last week we completed the assemblies of ROAR340 annotated them with Prokka, and started to explore the sequences visually using Artemis. Today, we are going to perform different types of analyses to answer a set of questions about the strains. Each group is assigned one strain and one question. Your poster presentation in the next workshop will be centred on answering the question you have been assigned.

Questions that different groups of students will try to answer:

**1. Is ROAR340 pathogenic?**

Tools to use:

• In Silico Clermont Phylotyper – identifies phylogenetic groups of *E. coli,*

• SerotypeFinder – identifies serotypes of *E. coli,*

• VirulenceFinder – predicts virulence factors in bacterial genomes,

• VFanalyzer– predicts virulence factors in bacterial genomes.

**2. Is ROAR340 antibiotic resistant? Is it resistant to any bacteriophage that might be used in phage therapy?**

Tools to use:

• ResFinder – identifies genes involved in antimicrobial resistance.

• Restriction-ModificationFinder – identifies restriction-modification system types in bacterial genomes,

• Prokaryotic Antiviral Defence LOCator (PADLOC) – identifies antiviral defence systems in bacterial genomes.

** The file you will be analysing in all of these tools is the SPAdes file called **'scaffolds.fasta'**

## Description of Bioinformatics tools

**Is ROAR340 pathogenic?**

### *In Silico* Clermont Phylotyper

A phylotype is a biological type that classifies an organism by its phylogenetic relationship to other organisms. *Escherichia coli* is composed of four main phylogenetic groups: A, B1, B2, and D. One of the methods of phylotyping (Clermont) is based on the presence or absence of the genes *TspE4, arpA, chuA*, and *yjaA*. In group A, only *arpA* is present; in group B1, *arpA* and *TspE4* are present; in group B2, *chuA, TspE4* and *yjaA* are present; in group D, only *arpA* and *chuA* are present. Some phylotypes are associated with virulent *E. coli* strains, for example, B2.

***URL*:** <https://ezclermont.hutton.ac.uk/>

***Reference*:** Beghain J, Bridier-Nahmias A, Le Nagard H, Denamur E, Clermont O. ClermonTyping: an easy-to-use and accurate in silico method for Escherichia genus strain phylotyping. Microb Genomics. 2018 Jul 1;4(7). doi: [10.1099/mgen.0.000192](https://doi.org/10.1099/mgen.0.000192)

***User instructions:***


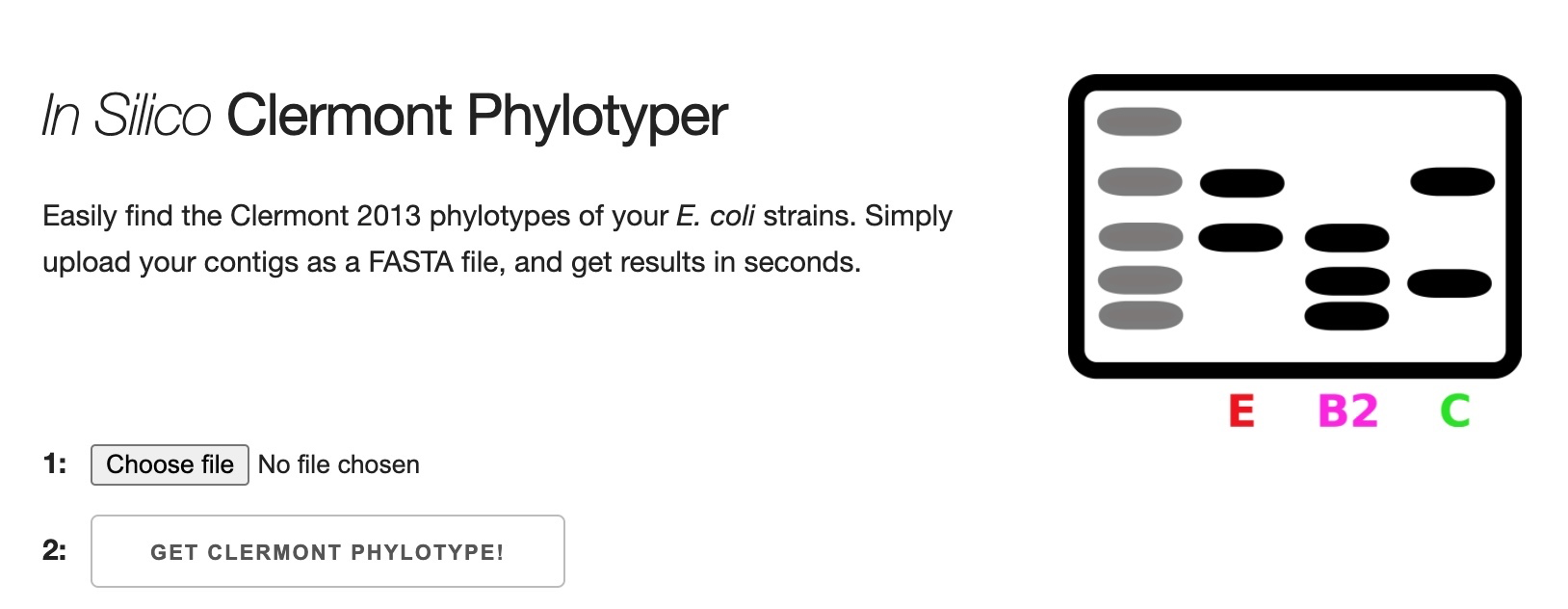


1. Press the “Choose a file” button and upload the ‘scaffolds.fasta’ file.
2. Press the “GET CLERMONT PHYLOTYPE!” button to process the uploaded file.

**Results**:

Results will be shown on the same page and include a profile for the strain (presence and absence of the genes *TspE4*, *arpA*, *chuA*, *yjaA*) and the final phylotype based on found genes.


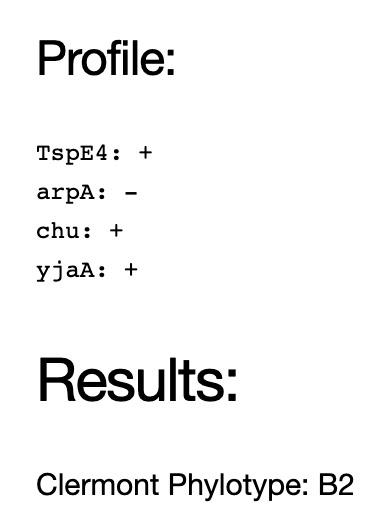


### SerotypeFinder 2.0

A serotype is a term that is used to refer to a group of organisms within a species that have the same type and number of surface antigens. For *E. coli*, O:K:H serotyping is based on a combination of the three immunogenic structures: the lipopolysaccharide (LPS) (O antigen), the capsular antigen (K), and the flagellar (H) antigen.

***URL***: <https://cge.food.dtu.dk/services/SerotypeFinder/>

***Reference***: Joensen KG, Tetzschner AMM, Iguchi A, Aarestrup FM, Scheutz F. Rapid and Easy In Silico Serotyping of Escherichia coli Isolates by Use of Whole-Genome Sequencing Data. Carroll KC, editor. J Clin Microbiol. 2015 Aug;53(8):2410–26. doi: [10.1128/JCM.00008-15](https://doi.org/10.1128/JCM.00008-15)

***User instructions:***


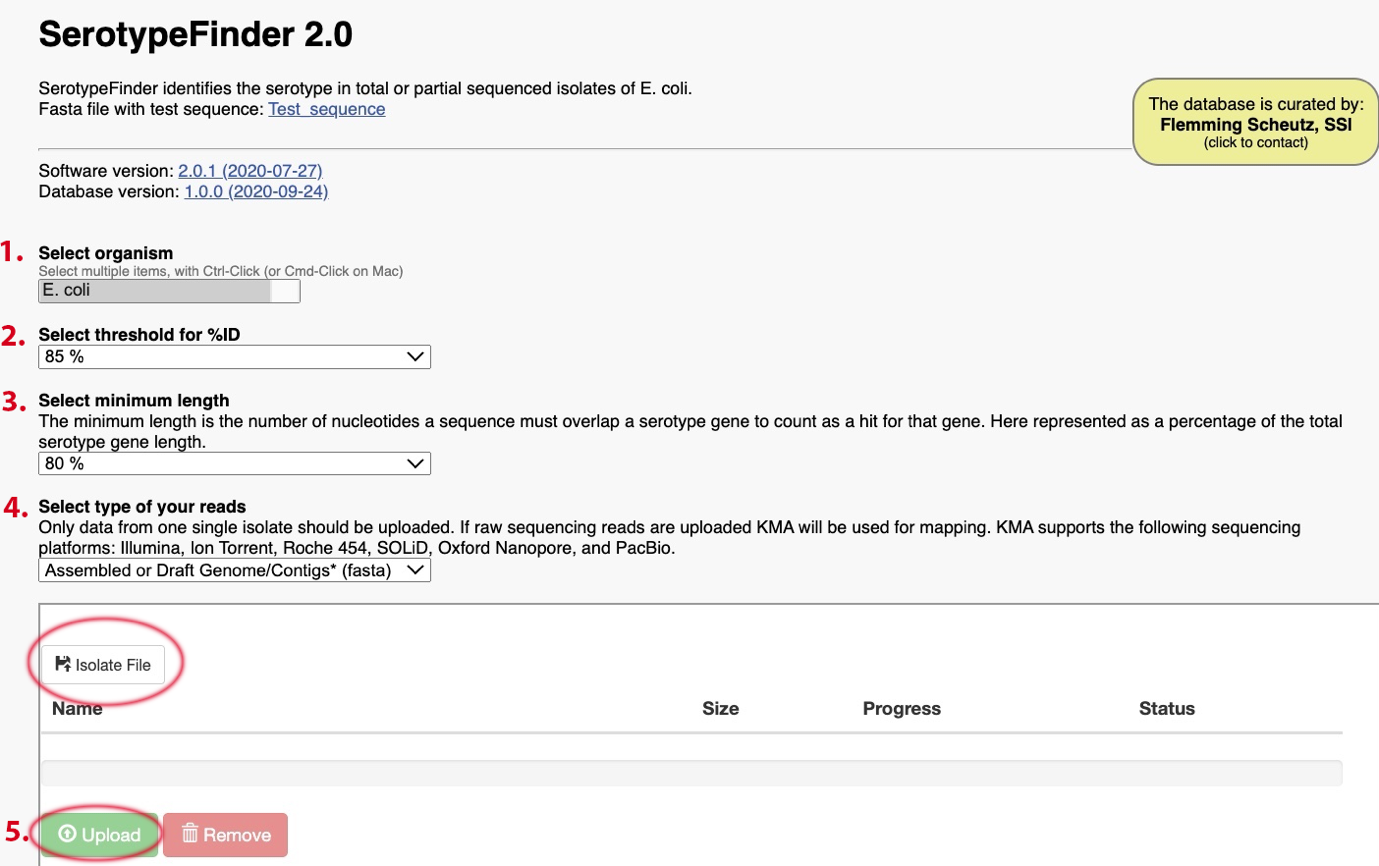


1. For the field “*Select organism*”, choose E. coli from the drop-down list.
2. To choose the minimum percentage of nucleotides that are identical between the best matching serotype gene in the database and the corresponding sequence in the genome, set the value “*Select threshold for %ID*” from the drop-down list, for example, 85%. You can select a higher or lower threshold to see variability in the number of matching serotype genes.
3. Select the minimum length for the gene overlap in the “Select minimum length” field, for example, 80%. You can select a higher or lower minimum length to see variability in the number of matching serotype genes.
4. Choose the file format of your data in the “Select type of your reads” field, for example, Assembled or Draft Genome/Contigs*, and press ‘Isolate File’. Next, choose the 'scaffolds.fasta' file and press “Open”.
5. To Upload your file, press the “Upload” button and wait for the results.

***Results:***

On the next screen with the message ‘Your job is being processed’, you can add your email to the field ‘To get notified by email’ and press the button ‘Notify me via email’, or you can keep the page open to get the results.

The results will contain two tables: “Database for H type genes” (a list of found H-antigen genes related to bacterial flagella) and “Database for O type genes” (discovered O-antigen genes associated with the outermost part of bacterial lipopolysaccharide (LPS)). The tables also contain determined serotype (column ‘Serotype’), % of identity (‘Identity’), and contig data on the position of the found gene ('Contig’, ‘Position in contig’ columns).


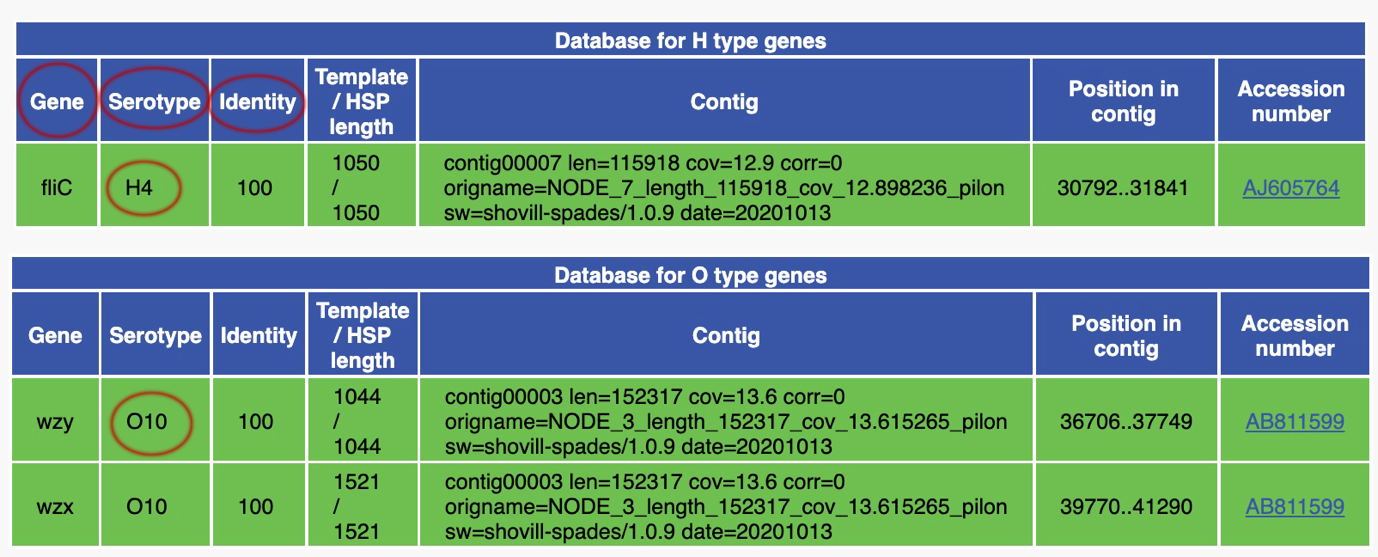


The detailed instructions are also provided at <https://cge.food.dtu.dk/services/SerotypeFinder-2.0/instructions.php>. The results explanation - at <https://cge.food.dtu.dk/services/SerotypeFinder-2.0/output.php>.

*Note*: SerotypeFinder 2.0 does not identify **capsular antigens.** They can be found using virulence factors tools VirulenceFinder and VFanalyzer.

## VirulenceFinder

***URL***: <https://cge.food.dtu.dk/services/VirulenceFinder/>

***Reference***: Joensen KG, Scheutz F, Lund O, Hasman H, Kaas RS, Nielsen EM, et al. Real-Time Whole- Genome Sequencing for Routine Typing, Surveillance, and Outbreak Detection of Verotoxigenic Escherichia coli. Carroll KC, editor. J Clin Microbiol. 2014 May;52(5):1501– 10. doi: [10.1128/JCM.03617-13](https://doi.org/10.1128/JCM.03617-13).

***User instructions:***


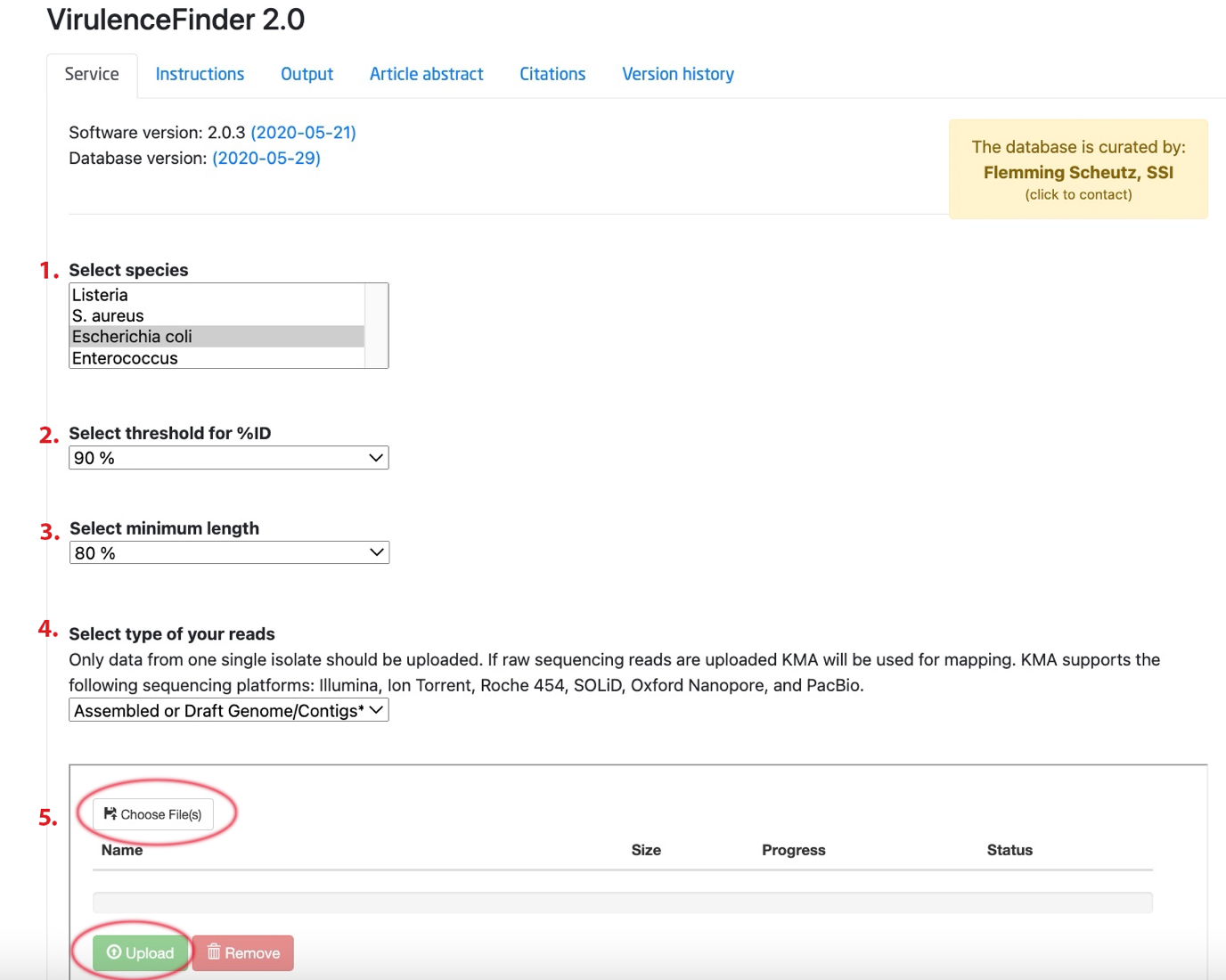


1. For the field “*Select species*”, choose Escherichia coli from the drop-down list.
2. To choose the minimum percentage of nucleotides that are identical between the best matching virulence gene in the database and the corresponding sequence in the genome, set the value “*Select threshold for %ID*” from the drop-down list, for example, 90%. You can select a higher or lower threshold to see variability in the number of matching virulence genes.
3. Select the minimum length for the gene overlap in the “Select minimum length” field, for example, 80%. You can select a higher or lower minimum length to see variability in the number of matching virulence genes.
4. Choose the file format of your data in the “Select type of your reads” field, for example, Assembled or Draft Genome/Contigs*, and press ‘Choose File’. Next, select the 'scaffolds.fasta' file and press “Open”. You can select a higher or lower minimum length to see variability in the number of matching serotype genes.
5. To Upload your file, Press the “Upload” button and wait for the results.

***Results:***

On the next screen with the message ‘Your job is being processed’, you can add your email to the field ‘To get notified by email’ and press the button ‘Notify me via email’, or you can keep the page open to get the results.

The results will contain two tables: a table with shiga-toxin genes and with virulent factors found in the uploaded genome. The column ‘Virulence factor’ contains found in the genome/contigs potentially virulent genes. The ‘Identity’ and ‘Query/Template length’ columns display the similarity to the database gene (a link to the gene is in the ‘Accession number’ column). The column ‘Protein function’ provides a short description of the protein expressed from the found virulent gene.


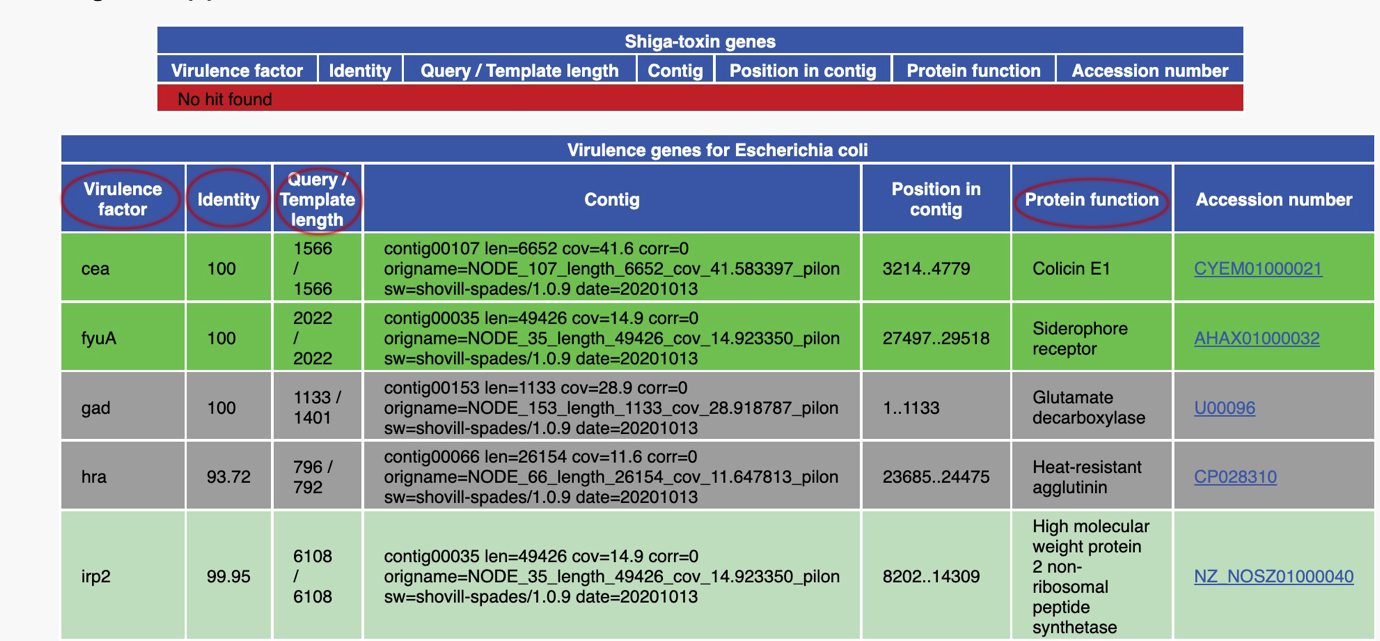


*Note*: Not all virulence factors are related to the pathogenicity of a bacterial strain, some just indicate an improved fitness of the strain.

The detailed instructions are also provided at <https://cge.food.dtu.dk/services/VirulenceFinder/instructions.php>. The results explanation - at <https://cge.food.dtu.dk/services/VirulenceFinder/output.php>.

## VFanalyzer

***URL*:** [**http://www.mgc.ac.cn/cgi-bin/VFs/v5/main.cgi?func=VFanalyzer**](http://www.mgc.ac.cn/cgi-bin/VFs/v5/main.cgi?func=VFanalyzer)

***Reference*:** Liu B, Zheng D, Jin Q, Chen L, Yang J. VFDB 2019: a comparative pathogenomic platform with an interactive web interface. Nucleic Acids Res. 2019 Jan 8;47(D1):D687–92. Available from https://academic.oup.com/nar/article/47/D1/D687/5160975. doi: [10.1093/nar/gky1080](https://doi.org/10.1093/nar/gky1080).

***User instructions:***


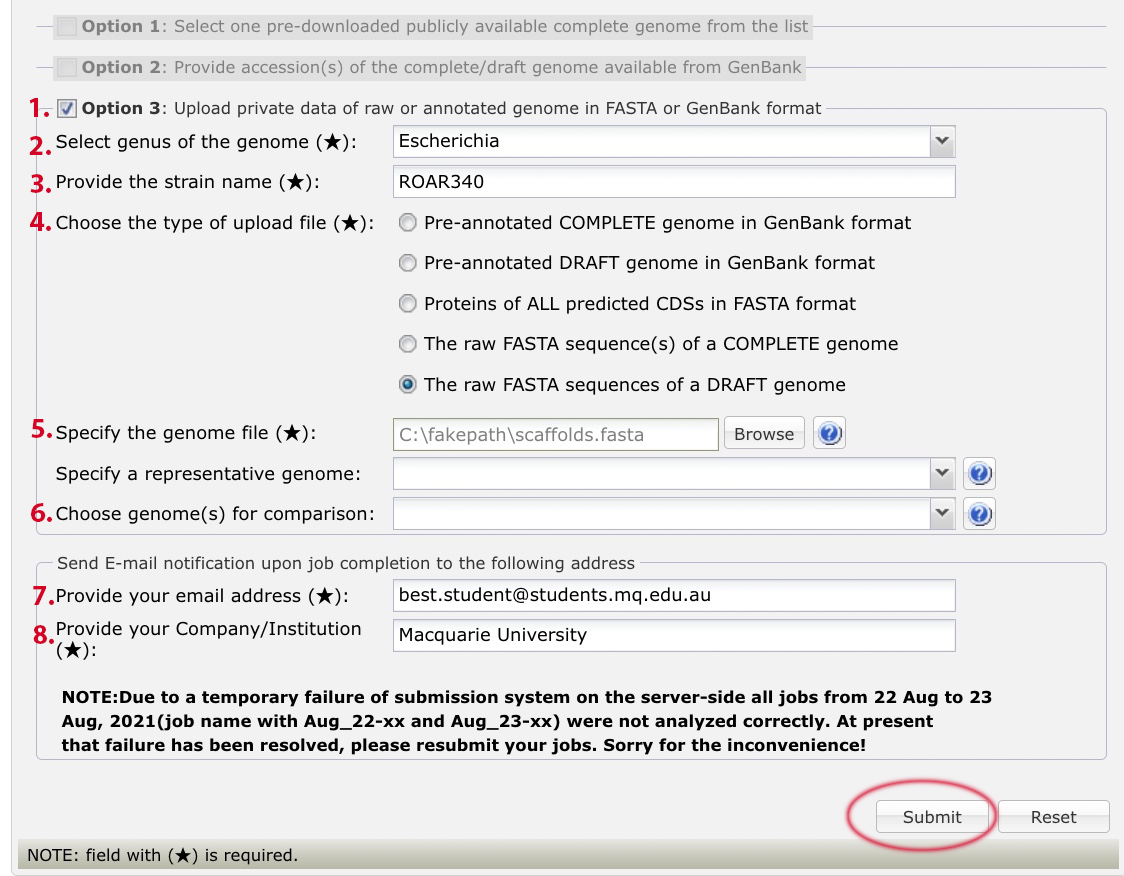


1. On the right side of the screen, select the “Option 3” check box.
2. Select genus of the genome: “Escherichia”.
3. Provide the strain name: for example, ROAR 340.
4. Choose the file type: “The raw FASTA sequences of a DRAFT genome”.
5. Specify the genome file: upload the ‘scaffolds.fasta’ file. Specify a representative genome - leave empty.
6. Choose genome(s) for comparison: leave empty
7. Provide your email address: your email address for the results notification
8. Provide your Company/Institution: add your university and press the “Submit” button. Wait until you receive a job ID (for example, Feb_18-4104320002). Copy the job ID and save it in your notes application.

***Results****:*

Results can be retrieved by using the form at the bottom of the screen or via a link in the email notification:

To retrieve results via the form, insert your saved job ID in the form and press the magnifier button.
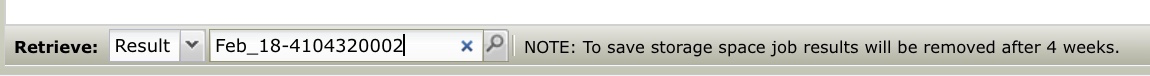
 As it takes around 20-30 min to get the results, it is more convenient to wait for the email notification. The result page contains a table with all found virulence factors ('Virulence factors’ column) and related genes ('Related genes’ column).

A comparison with the closest pathogenic strains is also provided on the right side of the result table. Pathogenic *E.coli* strains might cause enteric/diarrhoeal disease, urinary tract infections (UTIs) and sepsis/meningitis. In the result page provided below, the comparison is shown with UPEC (uropathogenic), and NMEC (neonatal meningitis) strains that belong to the group of extraintestinal *E. coli* pathogens.


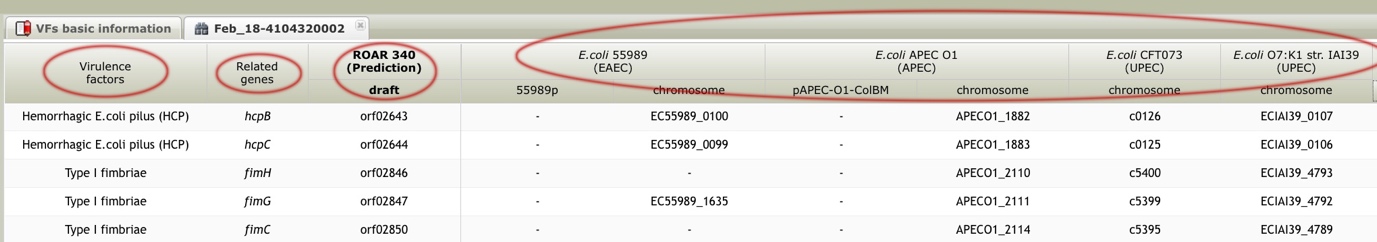


*Note*: Not all virulence factors are related to the pathogenicity of a bacterial strain, some just indicate an improved fitness of the strain.

**Is ROAR340 antibiotic resistant? Is it resistant to any bacteriophage that might be used in phage therapy?**

## Restriction-Modification Finder

Bacterial restriction-modification (R-M) systems cleave foreign DNA in specific recognition sequences. The systems include a restriction endonuclease enzyme that can cleave unmethylated foreign DNA at specific recognition sequences and a methyltransferase that methylates native DNA, preventing it from being cut at the same recognition sequence. Four types of R-M systems are known in *E. coli* (EcoKI, EcoRI, EcoP1, and EcoMcrBC), that differ in recognition site, cleavage position, subunit composition, and cofactor requirement.

***URL***: <https://cge.food.dtu.dk/services/Restriction-ModificationFinder/>

***Reference***: Roer L, Hendriksen RS, Leekitcharoenphon P, Lukjancenko O, Kaas RS, Hasman H, et al. Is the Evolution of Salmonella enterica subsp. enterica Linked to Restriction-Modification Systems? Eisen J, editor. mSystems. 2016 Jun 28;1(3). doi: [10.1128/mSystems.00009-16](https://doi.org/10.1128/mSystems.00009-16)

***User instructions:***


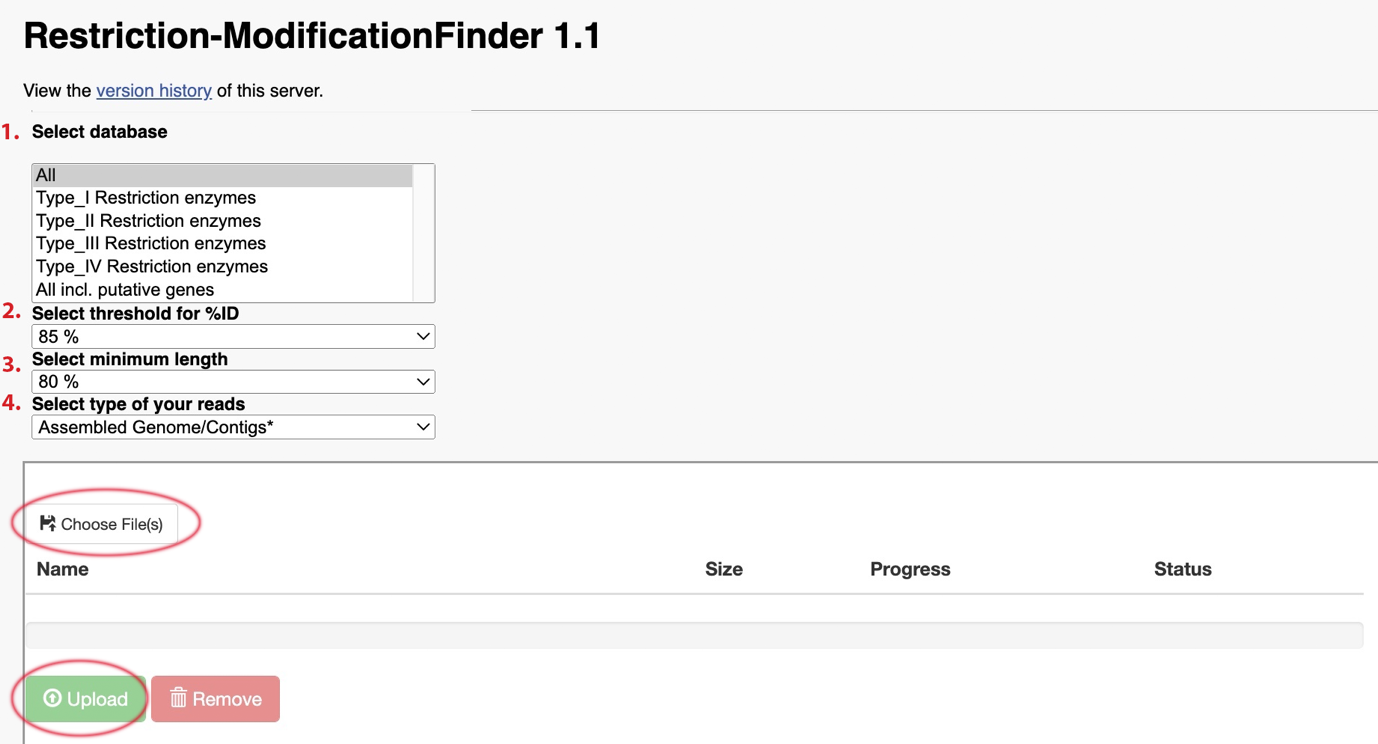


1. Select “All” in the field “*Select database*”.
2. To choose the minimum percentage of nucleotides that are identical between the best matching gene in the database and the corresponding sequence in the genome, set the value “*Select threshold for %ID*” from the drop-down list, for example, 85%. You can select a higher or lower threshold to see variability in the number of matching genes.
3. Select the minimum length for the gene overlap in the field “Select minimum length”, for example, 80%. You can select a higher or lower minimum length to see variability in the number of matching genes.
4. Choose the file format of your data in the “Select type of your reads” field as “Assembled or Draft Genome/Contigs*”, and press ‘Choose File(s)’. Next, select the 'scaffolds.fasta' file and press “Open”.
5. To Upload your file, Press the “Upload” button and wait for the results.

***Results*:**

The Results page contains all found restriction-modification systems among Type I, Type II, Type III, and Type IV.


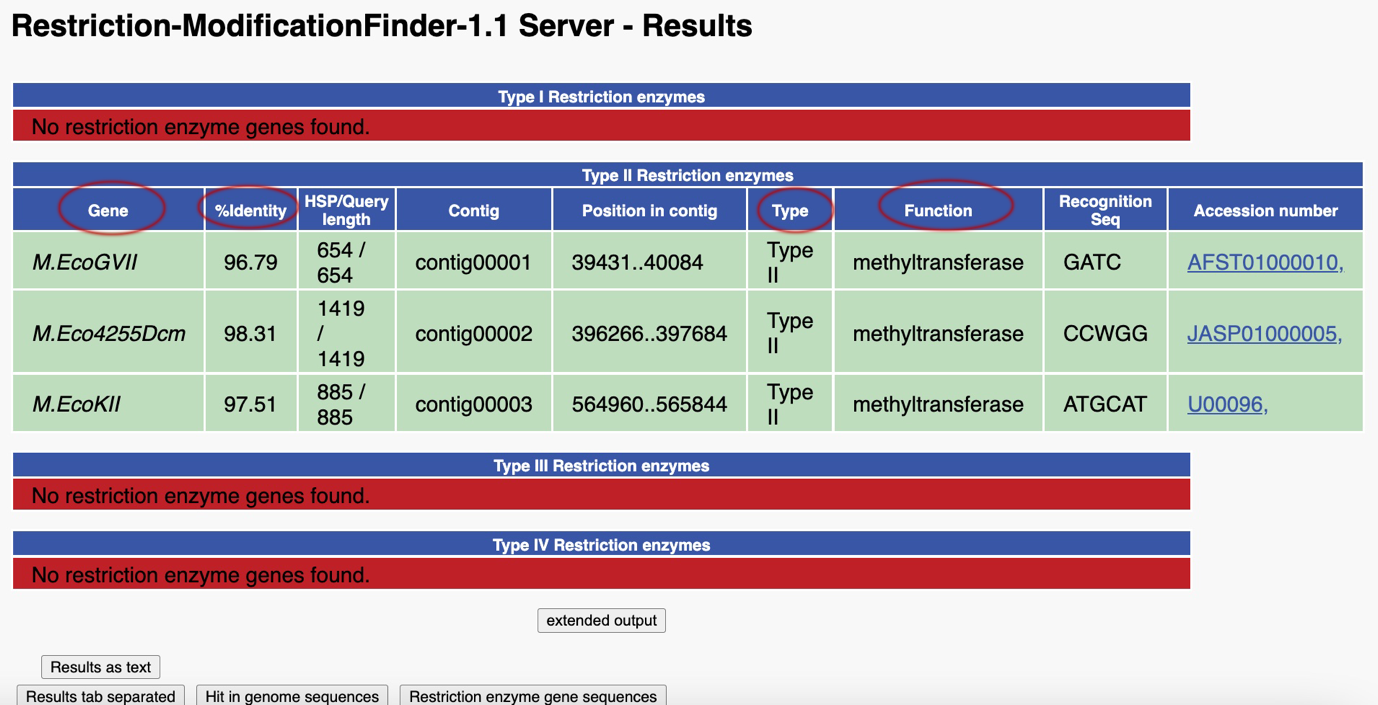


*Note*: The tool can show separate genes that belong to a particular R-M system, but they might not form the complete R-M system.

The detailed instructions are also provided at <https://cge.food.dtu.dk/services/Restriction-ModificationFinder/instructions.php>. The results explanation - at <https://cge.food.dtu.dk/services/Restriction-ModificationFinder/output.php>.

## Prokaryotic Antiviral Defence LOCator (PADLOC)

The tool identifies anti-phage defence systems in bacterial genomes, including CRISPR-Cas and additional defence systems such as CBASS, Druantia, Gabija, Hachiman, Kiwa, BREX, DISARM, and others.

***URL***: <https://padloc.otago.ac.nz/padloc/>

***Reference***: Payne L.J., Todeschini T.C., Wu, Y., Perry B.J., Ronson C.W., Fineran P.C., Nobrega F.L. and Jackson S.A.# (2021)
Identification and classification of antiviral defence systems in bacteria and archaea with PADLOC reveals new system types.
Nucleic Acids Research **49**, 10868-10878. doi: [10.1093/nar/gkab883](https://doi.org/10.1093/nar/gkab883)

***User instructions*:**


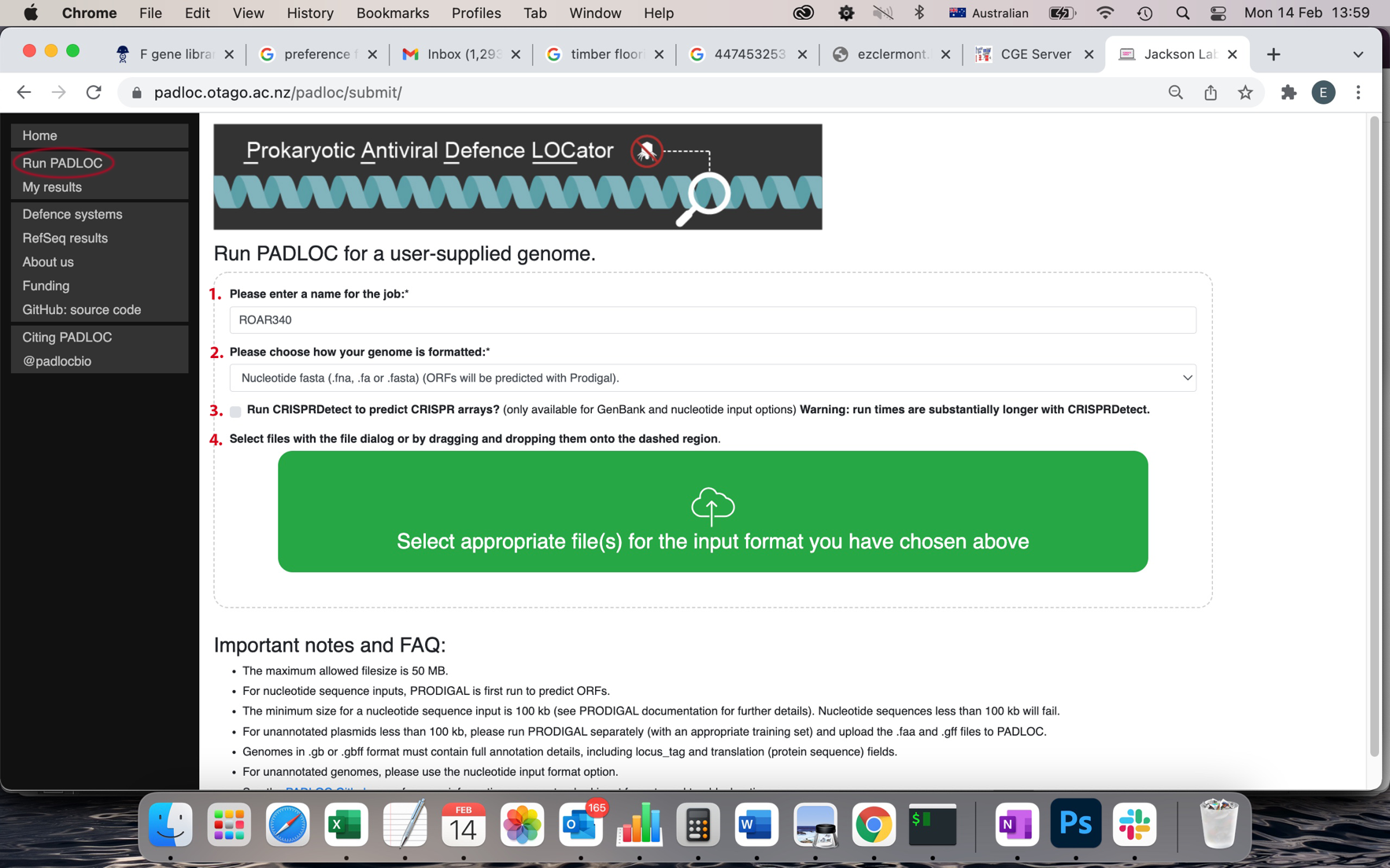


To start a new job, press the link “Run PADLOC” on the left-side menu.

On the next screen:

1. Enter a job name (for example, “ROAR340”).
2. Choose a file format (.fna, .fa, .fasta).
3. Deselect the “Run CRISPRDetect to predict CRISPR arrays” check box.
4. Upload the 'scaffolds.fasta' file.
5. After the file upload, press “Submit job”.


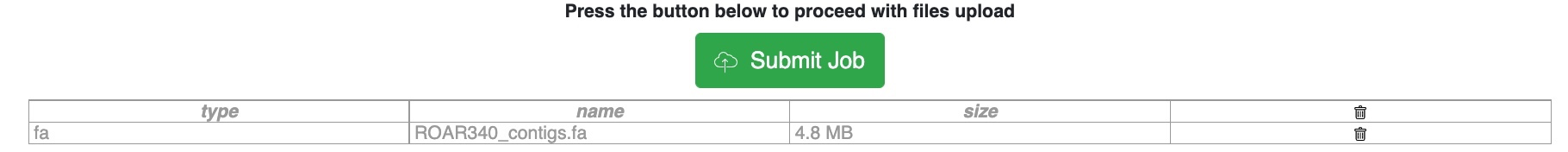


1. When a job is completed (its status is ‘Complete’), press on the job name to see the results.

***Results*:**

On the Results screen, press a link with your “Job name”. All found defence systems will be shown graphically (“Systems detected (image)”) and in the table (“Systems detected (simplified table)”). To download results in the CSV format, press the “Download .csv” button.


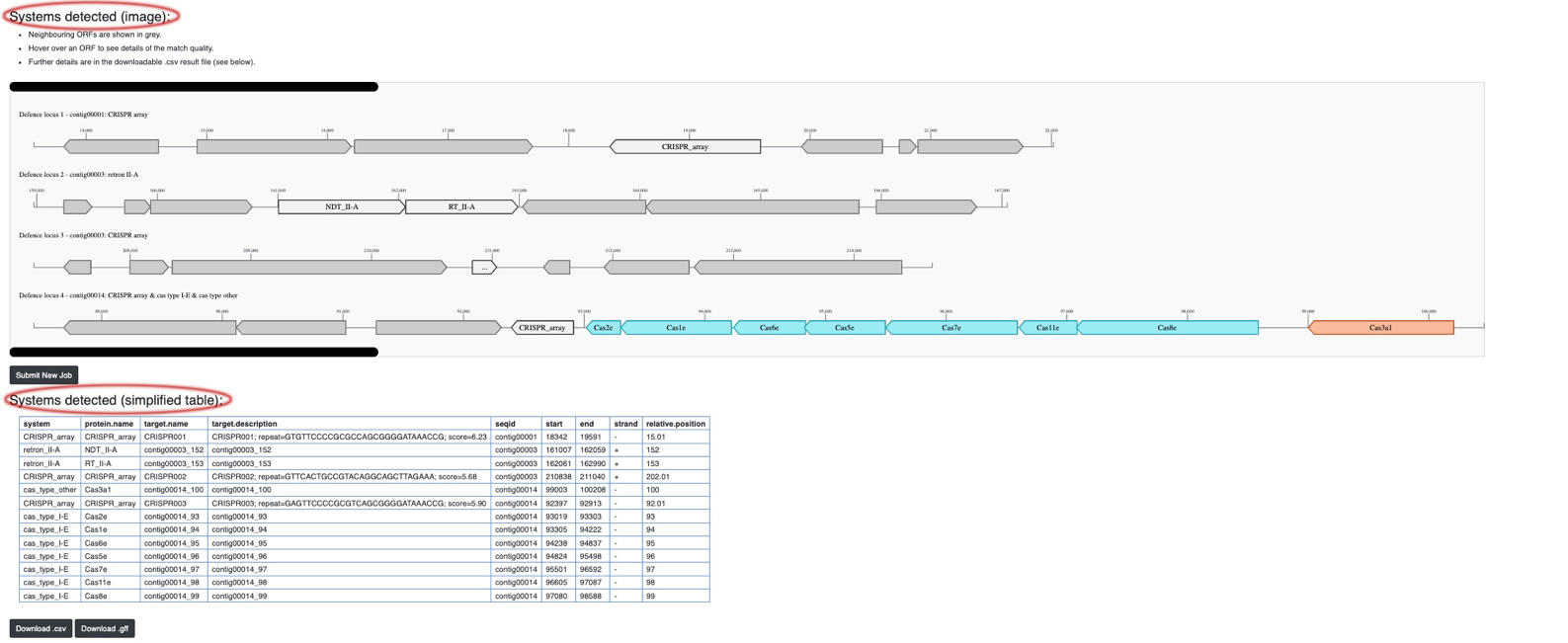


## ResFinder

ResFinder identifies acquired genes and/or finds chromosomal mutations mediating antimicrobial resistance in the total or partial DNA sequence of bacteria.

***URL***: <https://cge.food.dtu.dk/services/ResFinder/>

***Reference***: Bortolaia V, Kaas RS, Ruppe E, Roberts MC, Schwarz S, Cattoir V, et al. ResFinder 4.0 for predictions of phenotypes from genotypes. J Antimicrob Chemother. 2020 Dec 1;75(12):3491–500. doi: [10.1093/jac/dkaa345](https://doi.org/10.1093/jac/dkaa345)

***User instructions*:**


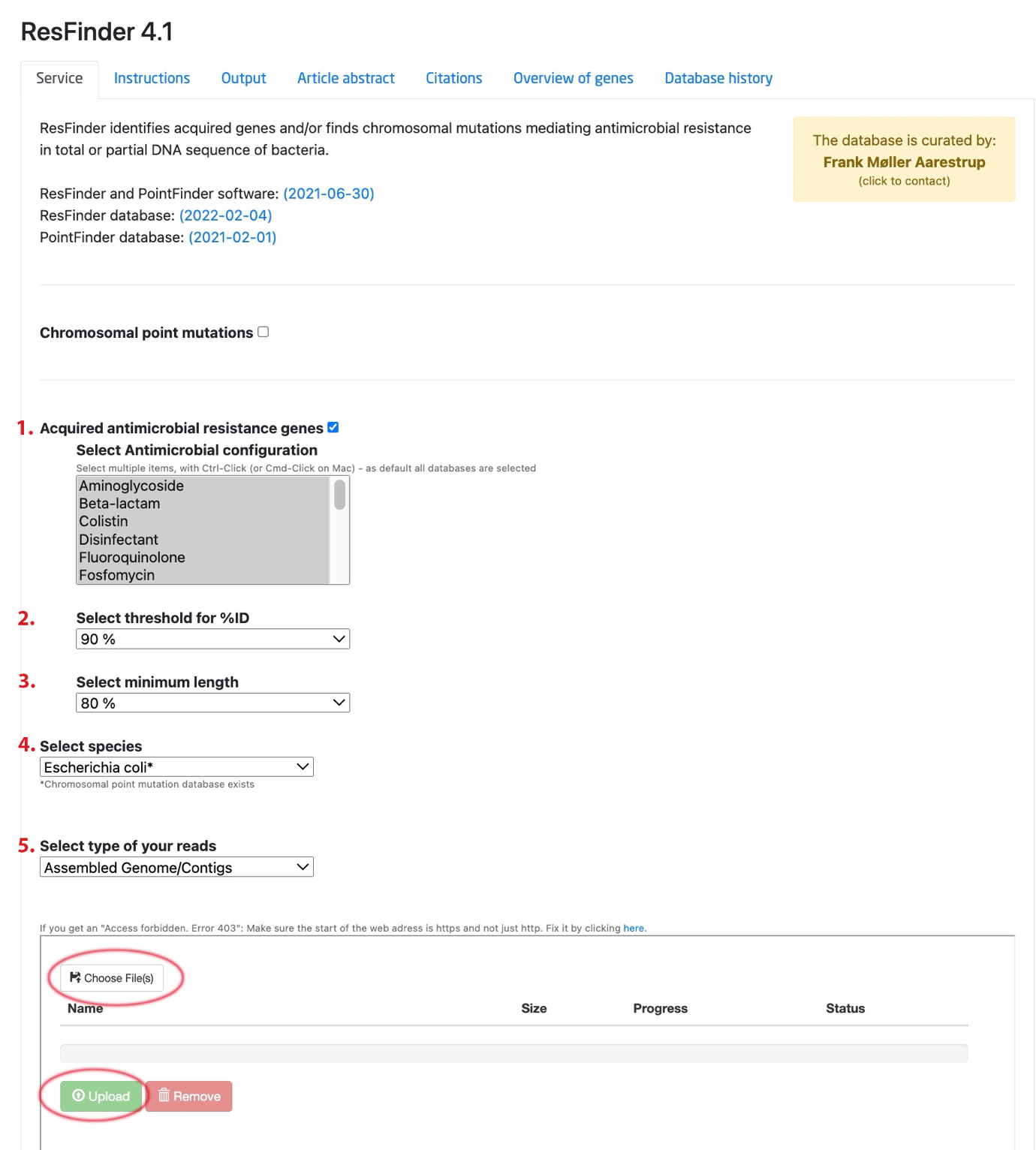


1. Click the “Acquired antimicrobial resistance genes” checkbox, and all available antibiotic classes will be selected.
2. To choose the minimum percentage of nucleotides that are identical between the best matching gene in the database and the corresponding sequence in the genome, set the value “*Select threshold for %ID*” from the drop-down list, for example, 90%. You can select a higher or lower threshold to see variability in the number of matching genes.
3. Select the minimum length for the gene overlap in the field “Select minimum length”, for example, 80%. You can select a higher or lower minimum length to see variability in the number of matching genes.
4. Select “Escherichia coli” in the “Select species” field.
5. Choose the file format of your data in the “Select type of your reads” field as “Assembled or Draft Genome/Contigs*” and press ‘Choose File(s)’. Next, select the 'scaffolds.fasta' file and press “Open”.
6. To Upload your file, Press the “Upload” button and wait for the results.

***Results*:**


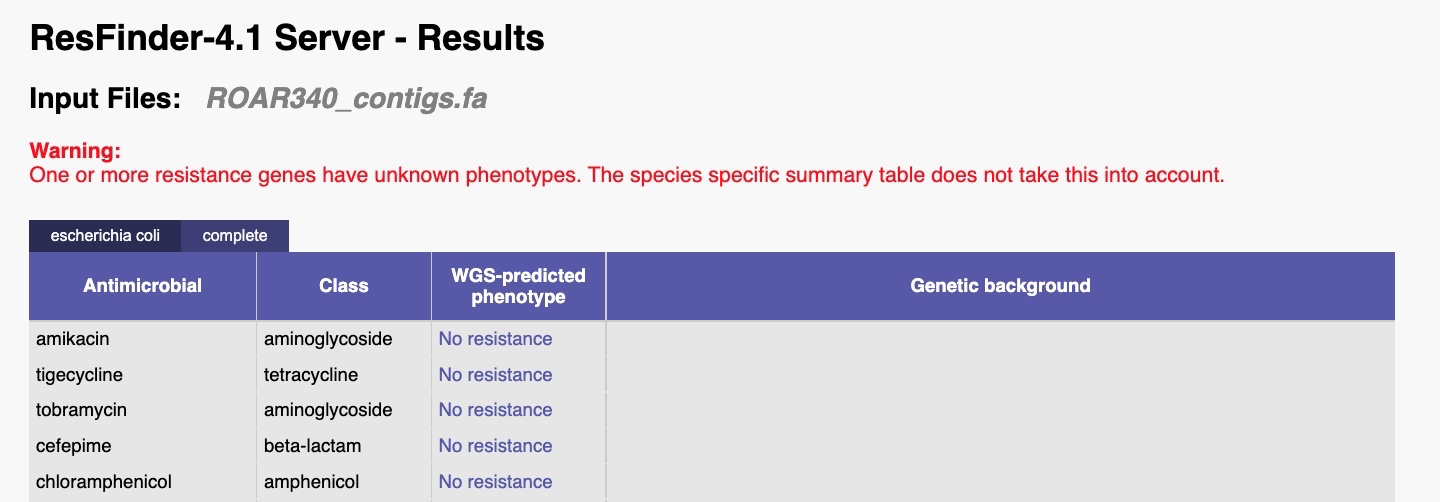


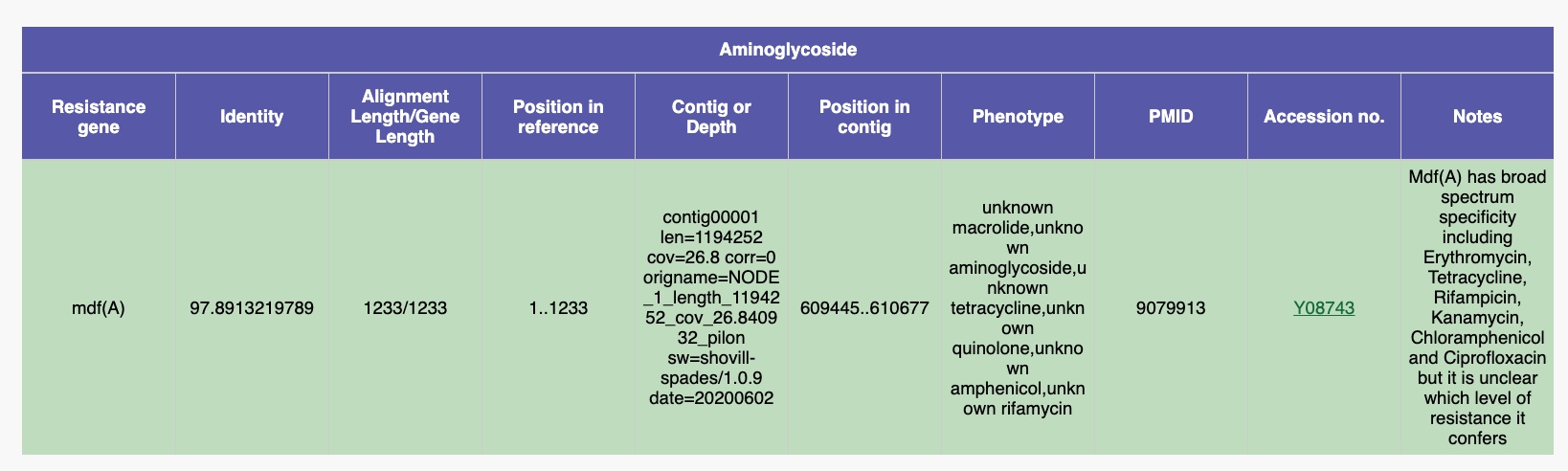


The Results page contains a table with all antimicrobials that were analysed (“Antimicrobial”), their class (“Class”), and if resistance was found or not (“WGS-predicted phenotype”).

Additional tables contain information on genes that might be involved in resistance to particular antimicrobial classes and include the gene name ("Resistance gene”), % of identity (“Identity”), gene length (“Alignment Length/Gene Length”), a contig that contains the gene ("Contig or Depth”), predicted phenotype based on the resistance gene (“Phenotype”), a link to reference genome sequence (“Accession no.”).

The detailed instructions are also provided at <https://cge.food.dtu.dk/services/ResFinder/instructions.php>. The results explanation - at <https://cge.food.dtu.dk/services/ResFinder/output.php>.
